# Structural basis of intron selection by U2 snRNP in the presence of covalent inhibitors

**DOI:** 10.1038/s41467-021-24741-1

**Published:** 2021-07-23

**Authors:** Constantin Cretu, Patricia Gee, Xiang Liu, Anant Agrawal, Tuong-Vi Nguyen, Arun K. Ghosh, Andrew Cook, Melissa Jurica, Nicholas A. Larsen, Vladimir Pena

**Affiliations:** 1 Research Group Mechanisms and Regulation of Splicing, The Institute of Cancer Research, London, UK; 2 H3 Biomedicine, Inc, Cambridge, MA USA; 3 Departments of Chemistry and Medicinal Chemistry, Purdue University, West Lafayette, IN USA; 4 Department of Molecular, Cell and Developmental Biology, University of California, Santa Cruz, CA USA; 5 Present Address: Cluster of Excellence Multiscale Bioimaging (MBExC), Universitätsmedizin Göttingen, Göttingen, Germany

**Keywords:** RNA splicing, Cryoelectron microscopy, X-ray crystallography

## Abstract

Intron selection during the formation of prespliceosomes is a critical event in pre-mRNA splicing. Chemical modulation of intron selection has emerged as a route for cancer therapy. Splicing modulators alter the splicing patterns in cells by binding to the U2 snRNP (small nuclear ribonucleoprotein)—a complex chaperoning the selection of branch and 3′ splice sites. Here we report crystal structures of the SF3B module of the U2 snRNP in complex with spliceostatin and sudemycin FR901464 analogs, and the cryo-electron microscopy structure of a cross-exon prespliceosome-like complex arrested with spliceostatin A. The structures reveal how modulators inactivate the branch site in a sequence-dependent manner and stall an E-to-A prespliceosome intermediate by covalent coupling to a nucleophilic zinc finger belonging to the SF3B subunit PHF5A. These findings support a mechanism of intron recognition by the U2 snRNP as a toehold-mediated strand invasion and advance an unanticipated drug targeting concept.

## Abstract (web summary)

Chemical modulation of intron selection has emerged as a route for cancer therapy. Here, structures of the U2 snRNP’s SF3B module and of prespliceosome- both in complexes with splicing modulators- provide insight into the mechanisms of intron recognition and branch site inactivation.

## Introduction

During splicing, introns are removed from the nascent transcripts by two transesterification reactions catalyzed by the spliceosome—a molecular machine composed of five ribonucleoprotein particles (snRNPs), known as U1, U2, U4, U5, and U6, and additional non-snRNP factors^1^. Spliceosomes assemble on introns by the stepwise recognition of conserved sequences that provide the reactive groups for catalysis: the 5′SS (splice site), the branch site (BS), followed downstream by the polypyrimidine tract (PPT) and the 3′SS. The spliceosome transits through several landmark complexes, such as E, A, B, C, P, and ILS, during the splicing pathway^1,2^.

The intron’s BS and 3′SS regions are defined early in the splicing cycle, in a process that often occurs in alternative ways^3^. The human BS sequence has a short and degenerate consensus motif YUNAY^4,5^, and is sometimes present in multiple, alternative copies selectively used in different cell types^6^. In yeast and subsets of human introns, BS and PPT-3′SS are recognized cooperatively by SF1 and the U2AF heterodimer, respectively^7–9^, whereas the U1 snRNP binds concomitantly to the 5′SS. These recognition events may occur across the intron (i.e., intron definition) or the exon (i.e., exon definition)^7,10,11^. Subsequently, displacement of SF1 enables the recruitment of the U2 snRNP—an intricate and dynamic apparatus essential for the accurate selection of the BS and 3′SS^4,5,12^. Following a currently unclear mechanism, the U2 snRNA component of the U2 snRNP base pairs to the BS in an ATP-dependent manner, yielding the prespliceosome A complex. The invariant BS adenosine (BS-A), extruded from the U2/BS helix, serves later as the reactive nucleophile during the branching reaction of splicing (reviewed in refs. ^13,14^).

The U2 snRNP has a bipartite organization, exhibiting a smaller U2 3′ module and a larger U2 5′ module^15–18^. The latter contains the heptameric SF3B complex, whose core subunit SF3B1 is recurrently mutated in cancers^15–19^. SF3B1 exhibits a HEAT domain (SF3B1^HEAT^) that adopts an open conformation in the 17S U2 snRNP and closed conformation in spliceosomes where it stabilizes the extended U2/intron helix and the polypyrimidine tract, in close proximity of the 3′SS^17,18,20–22^.

The protein–RNA and RNA–RNA rearrangements that enable selection of the BS and 3′SS by the U2 snRNP during the formation of prespliceosomes are complex and insufficiently understood^22–25^. Initially, the U2 snRNA is folded in a compact form within the 17S U2 snRNP, with the BS-interacting region presented as the loop region of the so-called branchpoint-interacting-stem-loop (BSL)^22,23^. After BS binding, the BSL should unwind and interact with the intron to form the extended U2/intron duplex, whose conformation remains unchanged throughout the A to B^act^ complexes^17,18^. The length of the helix is confined to 16 base pairs by a structural frame of the SF3B and SF3A proteins^17,18,26,27^. Concomitantly, SF3B1^HEAT^ accommodates the branch helix like a clamp, while trapping the BS-A within a pocket^17,18,20,21^. The BS-A binding pocket also serves as a hinge of the SF3B1 clamp (referred to as the hinged pocket). Simultaneously, SF3B1^HEAT^ binds the PPT several bases upstream of the 3′SS, suggesting an important contribution to the cooperative recognition of the BS and 3′SS^26,27^.

Pre-mRNA splicing defects have emerged as a hallmark for many cancers, and spliceosomes are viewed as promising therapeutic targets^28,29^. In particular, the selection of introns by the U2 snRNP is amenable to modulation by small-molecule antitumor compounds that share a common binding site on SF3B^28,30^. Referred to as splicing modulators, these compounds profoundly impact pre-mRNA splicing patterns in cells by inducing widespread exon skipping and intron retention in a manner that depends on the sequence of the inhibited substrate, modulators’ structure, affinity, and dose^30–33^. More than 20 modulators belonging to three distinct chemotypes—FR901464 analogs, herboxidienes, and pladienolides—have been reported, and some have entered clinical trials^28,29,32^. The first mechanistic insight into modulators’ binding to SF3B was provided by the recombinant SF3B core structure in a complex with pladienolide B (PB)^34^. The structure shows that the ligand occupies a hinged pocket in the open conformation of SF3B1, outcompeting the BS-A and preventing transition to the closed state of SF3B^20,32,35^. However, in the absence of native prespliceosome complexes stalled with modulators, the exact mechanism of interference in intron recognition remains unclear.

Identified more than two decades ago^36^, the splicing modulator FR901464 has a different chemistry than pladienolides and herboxidienes. Since then, several groups have proposed that the epoxide group carried by FR901464 analogs might react with SF3B’s subunits^37–40^. Extensively investigated in vitro and in vivo, the FR901464 analog spliceostatin A (SSA) provides a model system for splicing modulators and a molecular tool for dissecting the assembly of prespliceosomes and intron selection^31,41–43^. Intriguingly, SSA can recapitulate the effects of SF3B1 knockdown in cells^43^, suggesting a possible irreversible inactivation of SF3B1 through a yet unknown mechanism. However, at lower concentrations, FR901464 analogs have a less pleiotropic effect and inhibit splicing in a manner dependent on the intron sequence^31,33,42^.

In this work, by employing FR901464 analogs as molecular tools to dissect the splicing pathway, we elucidate how spliceostatins/sudemycins interfere with prespliceosome assembly and splicing commitment by covalent coupling to a reactive zinc finger of PHF5A. In addition, the structures support a general mechanism of intron selection by the U2 snRNP and provide a mechanistic explanation for the differential inhibition of introns in cells by known SF3B modulators.

## Results and discussion

### SSA arrests spliceosomes during the selection of introns

To elucidate the impact of antitumor splicing modulators on the splicing pathway and reveal how the U2 snRNP accurately selects and binds the introns, we set out to obtain the cryo-EM structure of a prespliceosome arrested by SSA. Attempting to avoid the potential intrinsic flexibility of cross-intron prespliceosomes^44^, we decided to assemble a U2-containing cross-exon complex^10^. We designed a model RNA substrate consisting of 54 intron nucleotides, the second MINX exon, the downstream 5′SS, and three MS2 aptamers for affinity purification (Supplementary Fig. 1a). This RNA construct is sufficient to recapitulate in vitro the effects of spliceostatins and pladienolides on spliceosome assembly, including dependence on ATP and a functional BS, as well as the sensitivity to heparin treatment^43^. Mass-spectrometric analysis of this cross-exon prespliceosome—referred to as the A3′ complex—reveals the presence of many proteins typically found in A complexes^45^. In contrast to cross-exon complexes purified in the absence of inhibitors^10^, the PRP5/DDX46 helicase (yeast Prp5p) is abundant, whereas the U4/U6.U5 tri-snRNP components are poorly represented in the A3′-SSA proteome (Supplementary Fig. 1b and Supplementary Data 1).

**Fig. 1:**
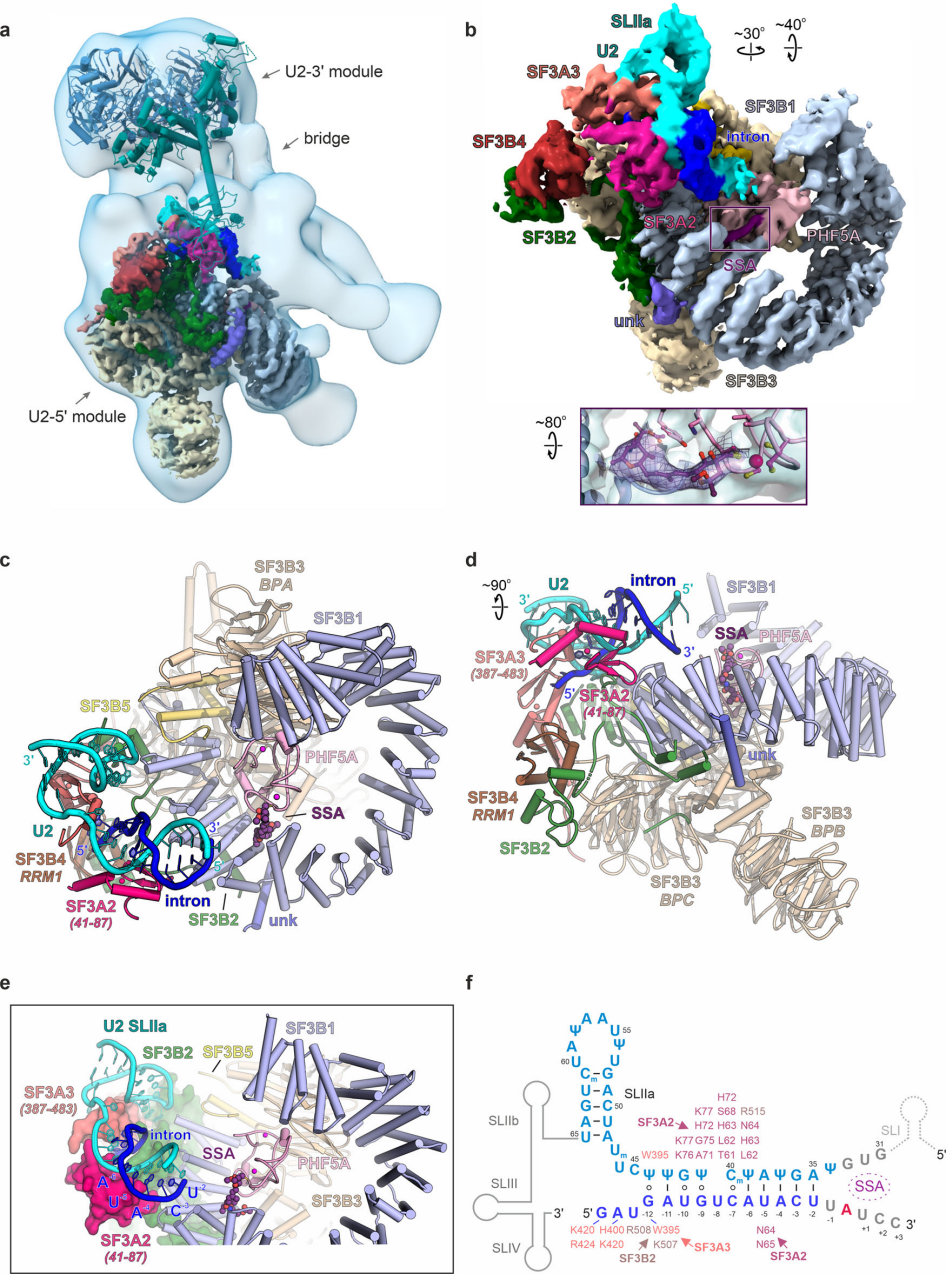
Cryo-EM structure of human A3′ prespliceosome-like complex arrested with SSA. **a** Overall cryo-EM density map of the U2 snRNP module of the A3′ prespliceosome. The density maps of the U2 5′ module and U2 snRNP are superimposed. The subunits are color-coded as in **b**. The proteins of the U2 3′ module and associated SF3A domains are docked as a rigid body and depicted in cartoon representation. **b** Cryo-EM density map of the U2 5′ module resolved at 3.1 Å resolution. The inset shows the density element observed in SF3B’s hinged pocket and corresponds to the SSA ligand. **c**, **d** Overall organization of the U2 5′ module in two orientations. **e** Structure of the U2 5′ module, emphasizing the U2/intron duplex relative to the surface of contacting subunits and the SSA. **f** Organization of the U2/intron structure within the A3′-SSA complex. Proteins and residues that interact with the U2/intron duplex are labeled. Methylated bases are marked with subscript “m”, and Ψ denotes pseudouridine. Except for **a**, all subunits are colored and labeled accordingly throughout the figure.

**Fig. 2:**
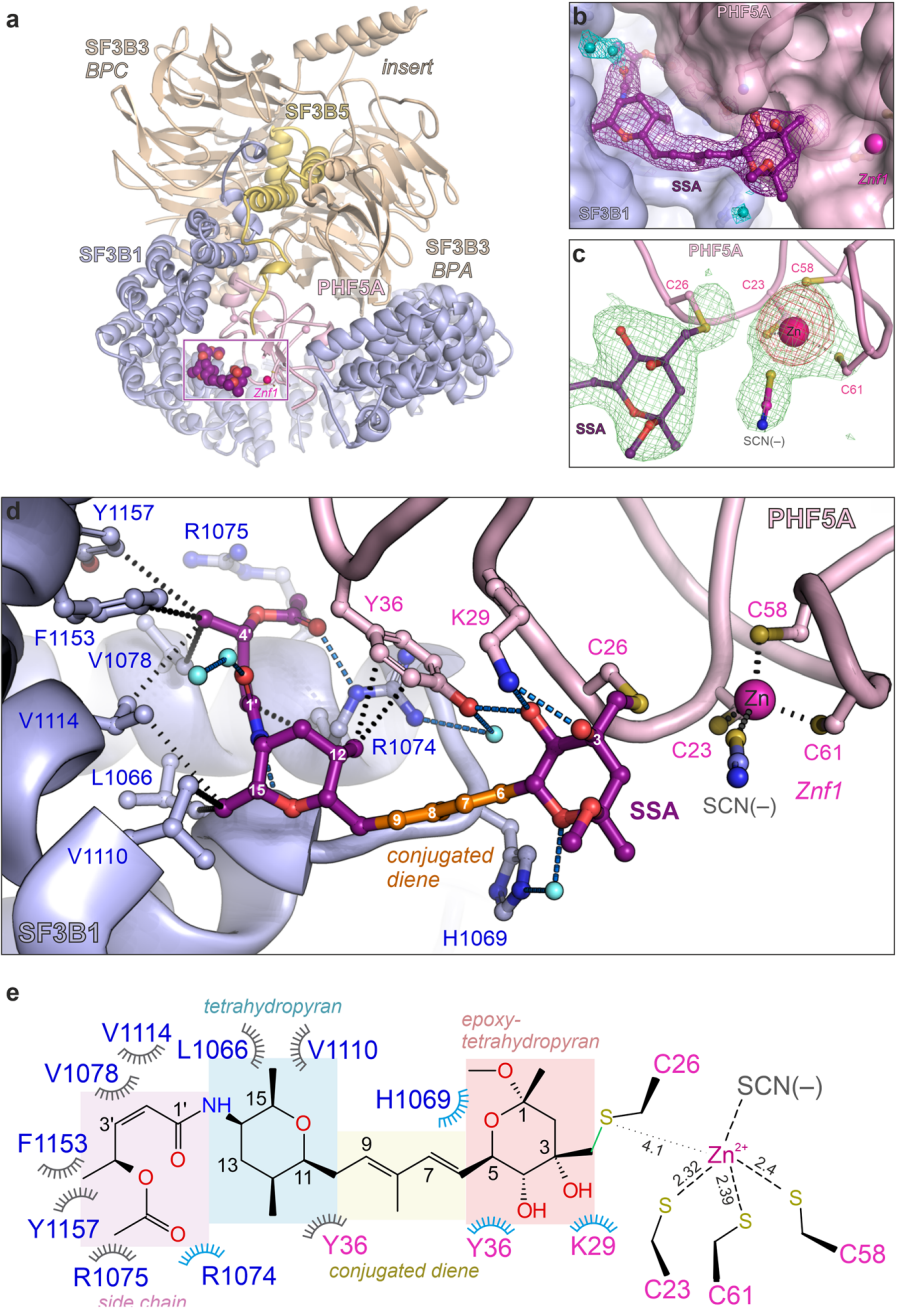
Crystal structure of SF3B in complex with SSA. **a** Structure overview of the SF3B^ΔBPB^ core in complex with SSA. **b** Electron density of the SSA within the crystal structure of the SF3B core. The 2*mF*o-*DF*c map is contoured at ~1.5 σ, and is displayed around the ligand and several water molecules (colored in purple and cyan, respectively). **c** Electron density (*mF*o-*DF*c, 3 σ) of the Znf1 motif of PHF5A and the covalently bound SSA ligand contoured around the final model. An anomalous difference map is depicted around the zinc ion and is colored red. **d** Crystal structure shows that SSA is engaged in multiple contacts with residues from the SF3B1-PHF5A binding tunnel. Distances between SSA and residues involved in hydrophobic contacts are shown as black dashes, whereas polar contacts are depicted as blue dashes. **e** Schematic depiction of the SSA interaction with residues from the hinged pocket, as observed in the crystal structure. Polar contacts are depicted in blue, whereas the hydrophobic interactions are colored gray. SSA’s functional groups are indicated as boxes.

Single-particle cryo-EM analysis of A3′-SSA enabled the reconstruction of a ~12 Å density map that corresponds to the U2 snRNP module of the complex. The overall map of the U2 snRNP shows two distinct lobes connected by a bridge where part of the SF3A complex is present (Fig. 1a and Supplementary Figs. 1c, d, 2). Focused classification and refinement enabled us to resolve the U2 5′ module to a resolution of ~3.1 Å (Supplementary Fig. 1e–h, Fig. 2 and Supplementary Table 1). In this map, we modeled the SF3B complex, the two matrin-type zinc-finger domains of SF3A2 and SF3A3, part of the intron substrate paired to the U2 snRNA, and the SSA ligand used to arrest the splicing reaction. SSA has an elongated, L-shaped density and is positioned in the hinged pocket of SF3B, between its SF3B1 and PHF5A subunits (Fig. 1b and Supplementary Fig. 3). We do not observe density for the DDX46 helicase from the 17S U2 snRNP, indicating either repositioning or destabilization during the intron’s pairing to U2 (Supplementary Fig. 4a, b). As expected, subunits and RNA structures specifically found in the 17S U2 snRNP (such as TAT-SF1 or U2 BSL) are not present in the A3′-SSA complex (Supplementary Fig. 4c–e). Surprisingly, SF3B adopts the same open conformation as in the 17S U2 snRNP or the isolated recombinant complex^20,22^, contrasting with the closed conformation observed in A-to-B^act^ spliceosomes (Supplementary Fig. 4f)^17,18,46^. Equally unexpected, U2 pairs with the intron, despite SSA having bound to the open state of SF3B. However, the U2/intron duplex observed in the presence of SSA, which we denote as the precursor U2/intron helix, is shorter than the extended U2/intron helix evidenced in the cross-intron A and later complexes^18^ and forms upon U2 pairing to the −12 to −2 intron region upstream of the BS-A. Importantly, the intron’s BS sequence has not fully paired U2, and the BS-A is not available for binding to SF3B’s hinged pocket, which is occupied by SSA (Fig. 1e, f).

Interestingly, an α-helix of unknown identity resides on the convex surface of SF3B1^HEAT^, on the opposite side of the hinged pocket that binds SSA (Fig. 1b–d). This location suggests a potential role of this helix in the transition from the open to the closed conformation. Furthermore, a globular density module that lacks discernable features is located at the periphery of the U2 snRNP (i.e., map M1, Supplementary Fig. 2a). It is currently unclear whether this density corresponds to the U1 snRNP or an assembly of non-snRNP proteins. Superposition of the cross-exon A3′ complex, the cross-intron yeast A complex, and the human pre-B complex shows that the unassigned density does not align to any other spliceosome subunits, including the U1 snRNP (Supplementary Fig. 5)^44,47^. Future studies are required to clarify this density’s possible relevance in the stepwise formation of cross-exon complexes.

### FR901464 analogs bind covalently to a reactive zinc finger of PHF5A

Despite the unambiguous localization of SSA by cryo-EM, the resolution is insufficient to explain in atomic details how the pocket recognizes SSA and the reasons for its irreversible effects in cells^43,48^. To resolve the small-molecule compound at a higher resolution, we set out to investigate its co-crystal structure with SF3B. We have engineered a variant of the human SF3B core that lacks the β-propeller BPB domain of SF3B3 (SF3B^ΔBPB^, Supplementary Fig. 6a, b). The complex formed crystals in two alternative space-groups, belonging to the orthorhombic and trigonal symmetry systems (Supplementary Fig. 6c–e and Supplementary Table 2). Our best orthorhombic crystals diffracted X-rays to a resolution of 2.3 Å, while diffraction data for the trigonal form was limited to ~3.0 Å.

The overall organization of the SF3B core and SSA’s conformation are largely the same as in the A3′-SSA complex (Fig. 2a)^34^. However, the level of detail is much higher at the 2.3 Å resolution (Fig. 2b–d and Supplementary Figs. 6f–i, 7). The distal half of the SSA molecule (C11-C15) establishes hydrophobic contacts and hydrogen bonds with the hinged pocket (Fig. 2d, e). The proximal half of SSA (atoms C1–C10) extends away from the tunnel towards one of the zinc fingers of PHF5A (i.e., Znf1, Fig. 2b). Its epoxy-tetrahydropyran group is recognized by a hydrogen bond network, involving SF3B1 and PHF5A residues and several solvent molecules in the binding pocket (Fig. 2d, e and Supplementary Fig. 7c, d). Strikingly, SSA’s epoxide group resides close to C26-PHF5A—one of the four cysteine residues of PHF5A’s Znf1 zinc cluster. The electron density is continuous between C26-PHF5A and the epoxide group, while absent between C26-PHF5A and Znf1’s zinc ion, indicating an intermolecular covalent bond between SSA and C26-PHF5A (Fig. 2c and Supplementary Fig. 6f–i). Although C26-PHF5A is no longer within coordination distance from the zinc ion, the anomalous difference density shows that the metal is not ejected from PHF5A upon covalent modification of Znf1. In turn, a water molecule appears to occupy the freed zinc coordination shell in the trigonal crystal form (Supplementary Fig. 8a).

Notably, a thiocyanate ion replaces the water molecule at Znf1 in the orthorhombic SF3B^ΔBPB^-crystals that grow in 200 mM potassium thiocyanate. The 2.3 Å resolution of these crystals enables visualization of thiocyanate’s distinctive elongated shape next to the zinc atom (Fig. 2c, d and Supplementary Figs. 6f, h, i, 7a, b, 8a). Importantly, crystals of SF3B^ΔBPB^ in complex with pladienolide D (PD) form as well in the presence of thiocyanate. However, no thiocyanate density is observed at the Znf1 cluster in SF3B^ΔBPB^-PD crystals (Supplementary Fig. 8a, b), consistent with the idea that the thiocyanate ion interacts with Znf1 only when a free zinc coordination shell is being made available (i.e., after the coupling reaction between SSA and C26-PHF5A has occurred).

**Fig. 3:**
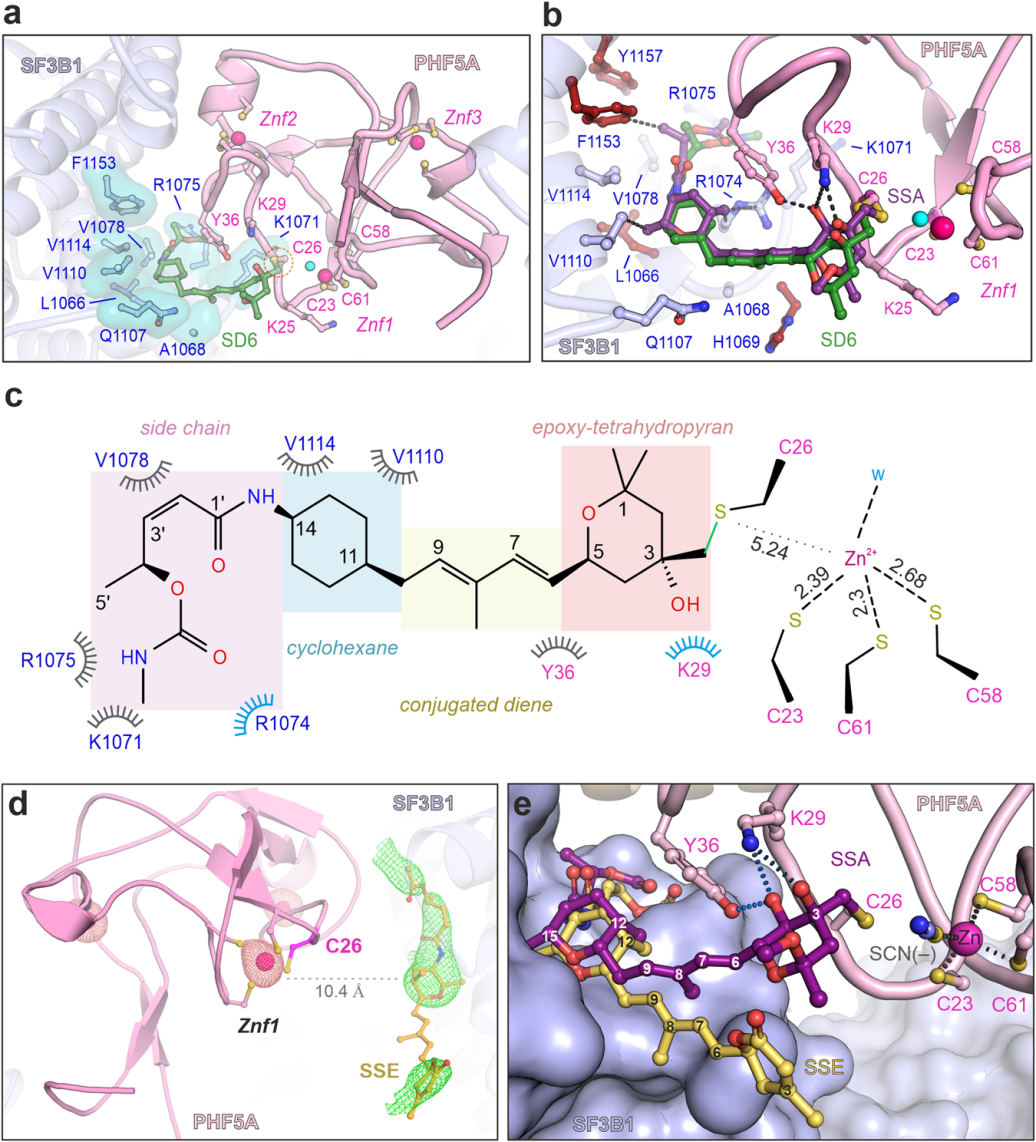
Crystal structures of SF3B in complex with SD6 and SSE. **a** Crystal structure shows that SD6 binds covalently to PHF5A and is engaged in contacts with fewer residues than those binding SSA (depicted and labeled). The water molecule and zinc ions are shown as cyan and magenta spheres, respectively. **b** Structural comparison between SSA and SD6 in co-crystal structure with the SF3B core. The interactions with chemical groups specific for SSA (i.e., absent from SD6) are shown. **c** Schematic depiction of the SD6 interaction with residues from the hinged pocket, as observed in the crystal structure. Polar contacts are depicted in blue, whereas the hydrophobic interactions are colored gray. SD6’s functional groups are indicated as boxes. **d** Crystal structure of SF3B^ΔBPB^ in complex with spliceostatin E (SSE). The polder (5.0 σ) electron density maps are displayed around SSE. An anomalous difference map (dark red, 6.0 σ) is contoured around PHF5A’s three zinc ions. Note the presence of ligand density in the SF3B1-PHF5A tunnel corresponding to the distal half of SSE. In contrast, density for the proximal moiety is largely absent, likely because of its flexibility in the absence of covalent coupling to C26-PHF5A. **e** Structural comparison between SSA and SSE in co-crystal structure with the SF3B core. Note that electron density was observed for the distal moiety of SSE, while the proximal moiety (C1–C10) is likely mobile. The depicted model is based on stereochemistry and space availability in the tunnel.

To investigate whether the covalent binding to PHF5A also occurs for the sudemycin analogs of FR901464^49,50^, we crystallized the SF3B^ΔBPB^ core in complex with sudemycin D6 (SD6, Fig. 3a, b)^33,51^. Like SSA, SD6 adopts the L-shaped pose, the thioether bond is present between the C13 atom of SD6 and C26-PHF5A, indicating that the pyran-epoxide moiety is a general feature of splicing modulators that bind covalently to SF3B (Fig. 3a–c and Supplementary Fig. 9, Supplementary Table 3). The reduced number of interactions between SD6 and the SF3B1-PHF5A pocked explains its lower potency and binding affinity compared to SSA^31,51,52^.

Spliceostatin E (SSE) lacks the reactive epoxide group, and previous studies indicate an inhibitory activity of several orders of magnitude lower than SSA in vitro^52^. To understand how an inactive SSA analog is recognized in the absence of the epoxide warhead group, we have also determined the structure of the SF3B^ΔBPB^–SSE complex at 3.0 Å resolution (Supplementary Table 3). The structure shows that the binding tunnel stabilizes the distal half of SSE, largely similar to the equivalent region of SSA and SD6. Conversely, the proximal half of SSE yields weak and noisy electron density, likely due to increased mobility caused by the lack of covalent coupling to C26-PHF5A (Fig. 3d and Supplementary Fig. 10). Comparison between the structures of SSA, SSE, and SD6 highlights a bipartite functional organization of FR901464 analogs, where the distal moiety is necessary and sufficient for molecular recognition of the tunnel, while the proximal moiety is required for the irreversible, covalent binding to the SF3B complex (Fig. 3e and Supplementary Figs. 8a, 10b,c).

### Reaction mechanism of covalent coupling

The epoxide rings are generally susceptible to attack from various nucleophiles, including thiols, especially when an appropriate base efficiently deprotonates the latter^53^. A notable precedent is provided by the antibiotic fosfomycin, which binds covalently to C115 residue from the catalytic center of the bacterial enzyme MurA. Although not belonging to a zinc finger, the nucleophilicity of the cysteine appears enhanced by the nearby guanidinium moieties of R120 and R397^54^.

**Fig. 4:**
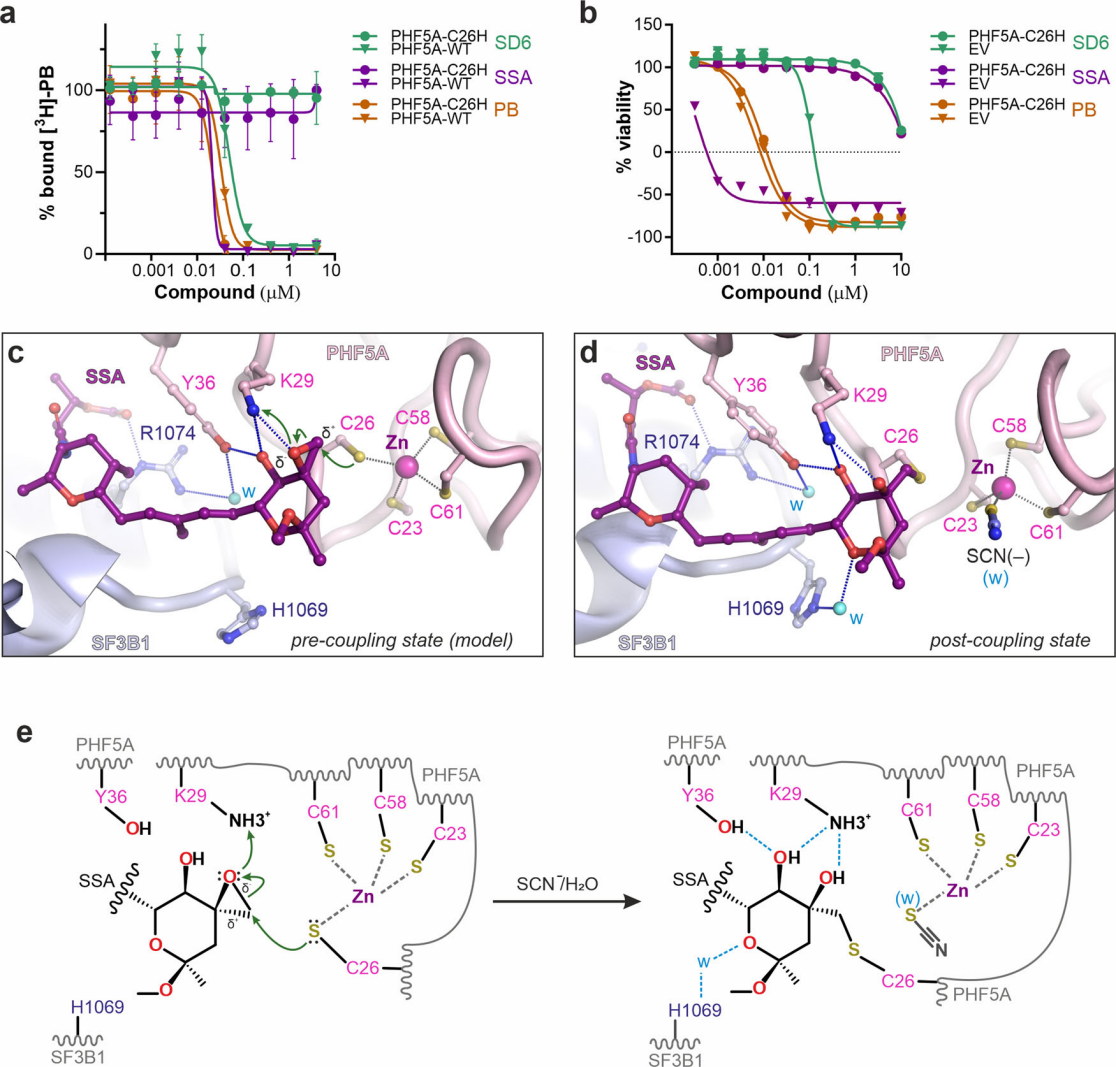
Mechanism of covalent coupling to the nucleophilic zinc finger. **a** Competitive titration of [^3^H]-pladienolide B (10 nM) with SSA, SD6, and PB by scintillation proximity assays (SPA). Error bar indicates SEM, *n* = 2 biological replicates. Source data are provided in the Source Data file. **b** Cell growth inhibition assays of HCT116 cells harboring the C26H-PHF5A point mutation. Control cells were transfected with an empty vector. Error bar indicates SEM, *n* = 2 biological replicates. Source data are provided in the Source Data file. **c** Model for the pre-coupling state was obtained by docking the unreacted ligand into the experimental density of the covalently-linked SSA. **d** Crystal structure of SF3B^ΔBPB^-SSA corresponding to the post-coupling state depicted as in **c** for a side-by-side comparison. **e** Mechanism of covalent coupling of epoxy modulators to the nucleophilic zinc finger of PHF5A, as inferred from crystal structures and stereochemistry. Available structural data and functional assays favor a nucleophilic substitution mechanism where the activated thiolate group of C26 carries the nucleophilic attack, whereas K29 stabilizes the leaving hydroxyl group. The zinc-bound C26 can be replaced either by water or thiocyanate.

However, covalent coupling between a zinc-coordinated cysteine and an epoxide ring is intriguing, as it was never reported for SSA, SD6, or other compounds. Therefore, we used several methods to show that covalent coupling is not dependent on the crystallization conditions. First, mass-spectrometric (MS) analysis of the SF3B core incubated with SSA or SD6 confirms that the molecular weight of detected PHF5A molecules increases with the expected mass of the bound modulators (Supplementary Fig. 11a). Secondly, we employed scintillation proximity assays with tritiated PB to show in vitro a dramatic loss of SSA and SD6 binding to the core SF3B complex harboring C26H-PHF5A (Fig. 4a). Third, we show that mutated cells harboring C26H-PHF5A can discriminate between covalent and non-covalent inhibitors, being insensitive to several orders of magnitude increase in SSA concentration. We do not observe this effect for PB, consistent with the critical importance of C26-PHF5A for the binding of covalent inhibitors to SF3B (Fig. 4b). In contrast, previously reported mutations of PHF5A (Y36A) or SF3B1 (R074H, V1078I/A) confer resistance both to pladienolide-related E7107 as well as SSA and SD6 (Supplementary Fig. 11c, d)^33^. As these residues are located remotely from the zinc finger (Figs. 2d, e and 3a, c), their substitutions are likely to impair the interactions with modulators’ distal moiety without interfering with the covalent coupling.

As captured in the crystal structure, the configuration of the chemical groups in the post-reaction state supports a nucleophilic substitution mechanism. The zinc atom serves a catalytic role in raising the nucleophilicity of C26-PHF5A by deprotonating the thiol group. The resulting thiolate anion attacks and opens the epoxy ring to form a thioether bond, while K29-PHF5A contributes to stabilizing the leaving group (Fig. 4c–e). Consistently, viability assays show that K29-PHF5A may have a mild impact on the coupling efficiency, with cells being less sensitive to SSA/SD6 when K29 was substituted for alanine or arginine (Supplementary Fig. 11b). Concomitantly, a water molecule likely replaces C26-PHF5A from the coordination sphere of the zinc atom, resulting in a configuration reminiscent of the active sites of zinc enzymes (e.g., carbonic anhydrase or alcohol dehydrogenase)^55^, where a water molecule and three side chains coordinate the zinc atom. Incidentally, thiocyanate substitutes the water in a crystal form that grows in the presence of this ion.

The proposed reaction chemistry bears some similarities with other systems, such as the Ada system in *E. coli*^56,57^ or the zinc ejectors compounds^56^ or fosfomycin coupling to MurA^53^. However, the covalent modification of PHF5A’s structural zinc-finger motif by spliceostatins/sudemycins is unique in its mechanism and shows how these modulators exploit the rich microenvironment of the SF3B binding tunnel to inactivate an early prespliceosome complex.

### Comparison between FR901464 and pladienolide analogs

**Fig. 5:**
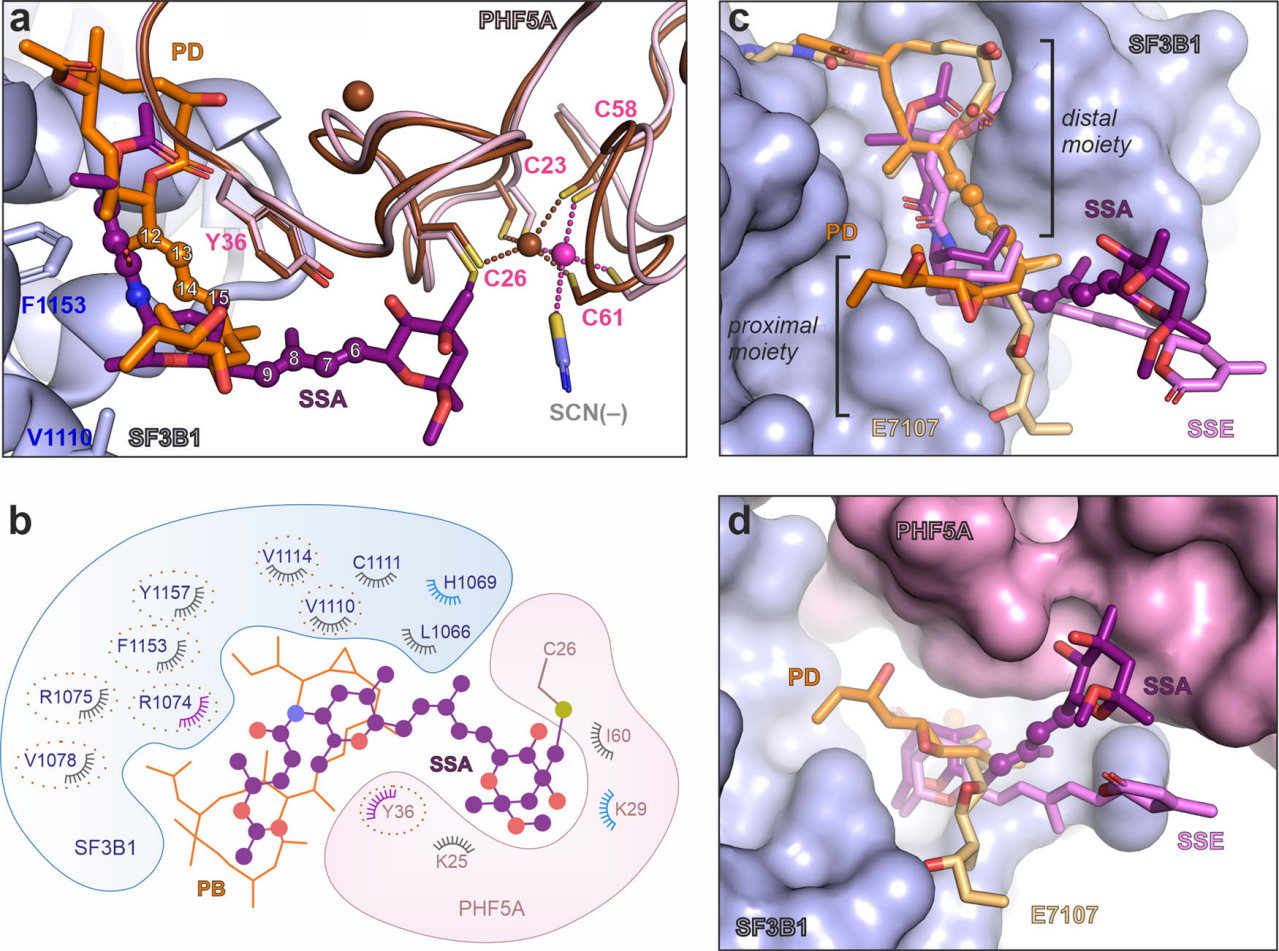
Structural comparison between covalent and non-covalent modulators. **a** Superposition of SF3B^ΔBPB^–SSA onto SF3B^ΔBPB^–PD. Atoms from the conjugated diene moiety are numbered. PHF5A complexed with SSA is colored brown. **b** Schematic of the SF3B1-PHF5A pocket with the PB (PDB 6EN4) and the SSA ligands superimposed. SF3B residues contacting SSA are indicated. Residues that also contact PB are circled. Residues involved in polar contacts and hydrophobic interactions with SSA are colored in blue and gray, respectively. **c** Comparison between SF3B^ΔBPB^–SSA, SF3B^ΔBPB^–SSE, and SF3B^ΔBPB^–PD structures. The crystal structure of isolated E7107 (DB12508) is superimposed on the macrolide ring of PD. PHF5A is not shown for clarity’s sake. **d** A different view of the superpositions shown in **c**. PHF5A is depicted in pink.

Although FR901464 and pladienolide analogs share the same binding pocket (Fig. 5)^34,52^, the determined structures highlight striking differences as well as unexpected similarities between these different families of compounds. First of all, this work establishes that FR901464 analogs are covalent inhibitors, in contrast to pladienolides. Despite this surprising difference, the existing structures suggest that both types of modulators bind SF3B in several steps, finally becoming stabilized in an L-shape pose induced by multiple constraints of the binding tunnel. One segment of the ligands’ L-shape follows a common binding path in FR901464 analogs and pladienolides, while the second segment is oriented in the opposite direction (Fig. 5a, b)^34^. Comparison between SSA, SD6, and SSE suggests their distal part initiates the molecular recognition by SF3B (Fig. 3a, e). In the next step, the warhead group belonging to the proximal moiety establishes polar and hydrophobic contacts with SF3B residues near PHF5A’s Znf1 motif, enabling the coupling reaction between the epoxy group and the thiolate moiety of C26-PHF5A (Fig. 4c–e).

Pladienolide derivatives appear to bind in two steps as well. Thus, a comparison between SF3B-PD^ΔBPB^, SF3B-PB and isolated E7107 suggests that pladienolides access the binding tunnel in an extended side-chain conformation^34,35^, while later bind stably by an induced fit reconfiguration of the aliphatic side-chain. This reconfiguration appears to “lock” the compound on SF3B, in the distinctive L-shaped conformation (Fig. 5a, c, d and Supplementary Fig. 8b). To some extent, the induced fit of the aliphatic side-chain and covalent coupling to Znf1 may serve similar roles for pladienolide derivatives and FR901464 analogs, respectively.

Contrary to earlier suggestions^57^, a striking difference between FR901464 analogs and pladienolides is the location and function of the conjugated diene—a distinctive moiety present in all families of modulators that bind SF3B^29,58^. In pladienolides, this moiety occupies the narrower and central part of the binding tunnel^34^. In covalent inhibitors, an amide group followed by a carbon-carbon double bond (C2′–C3′) replaces the role and location of the conjugated diene. (Fig. 5a, b). In contrast to pladienolides, the conjugated diene group in SSA and SD6 has a different function, serving as a rigid spacer that positions the epoxide group in the reach of PHF5A’s reactive zinc finger, while its contacts with the binding tunnel are minimal. Following these findings, the construction of spacers carrying epoxy warheads and their grafting on weakly binding compounds may guide the design of next-generation splicing modulators.

### A mechanism of intron’s progressive recognition by the U2 snRNA during prespliceosome formation

Often present in multiple copies within the same intron^6^, human BSs are to a great extent recognized before recruitment of the U2 snRNP to the intron^7–9^. The U2/intron duplex formation is likely essential in the BS’s final selection step for splicing commitment. Far exceeding the conserved YUNAY motif of BSs^4,5^, the extended U2/intron duplex consists of 16 base pairs in cryo-EM structures of A to B^act^ spliceosomes, confined by conserved residues of SF3A and SF3B complexes^17,18,26,27^. The duplex length is invariant between humans and yeast, suggesting that its formation mechanism is highly conserved.

**Fig. 6:**
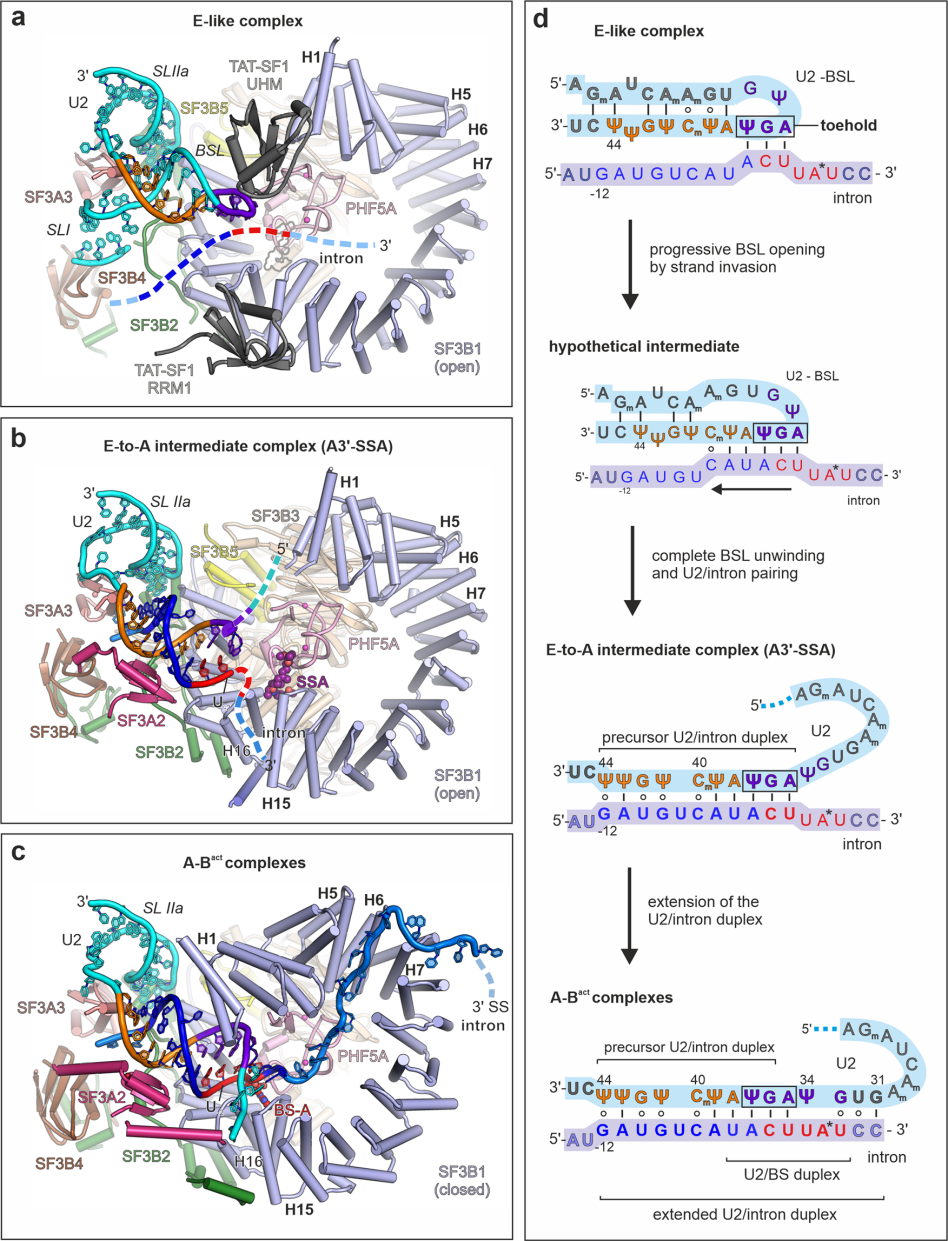
Mechanism of the stepwise intron selection by U2 snRNP. **a** The E-like complex model is based on the 17S U2 snRNP structure (PDB 6Y50), which is positioned in the proximity of an intron schematic. The equivalent location of SSA is shown as a silhouette, for orientation’s sake. **b** Structure of the U2 5′ module of the A3′ complex arrested with spliceostatin A (SSA). **c** Structure of the U2 5′ module from the A/B^act^ complexes. The depicted U2 5′ model is derived from the human B^act^ coordinates (PDB 5Z58). Complexes from **a** to **c** were superimposed over equivalent residues from PHF5A and SF3B1. **d** Schematic RNA transitions during intron’s recognition by U2, as suggested by cryo-EM structures. Nucleotides resolved in cryo-EM structures are shown in bold letters and colored as in **a**. Ψ and the “m” subscript denotes pseudouridine and methylated nucleobases, respectively. Throughout the entire figure, the BS consensus and nucleotides from BSL’s loop are shown in red and purple, respectively. The remaining sequence of the intron and of the U2 snRNA are colored in deep blue and orange, respectively. RNA strands not resolved in cryo-EM structures are dashed. All the other subunits and elements are color-coded as in Fig. 1.

The structure of the A3′-SSA captures an E-to-A intermediate, indicating how the U2/intron duplex forms in at least two steps (Fig. 6). Initial binding of the 17S U2 snRNP requires unwinding of the U2 BSL and pairing to the intron region −12 to −2 upstream of the BS-A. The resulting precursor—U2/intron duplex, resembles BSL with respect to length and orientation (Fig. 6a, b). Remarkably, the last three base pairs of the precursor originate in the BSL’s loop, consistent with previous observations^22,23^, indicating that the intron may establish its first contacts with BSL’s loop region (A35-U37). It would then invade the BSL’s stem asymmetrically, displacing the 5′-terminal moiety of the U2 snRNA from SF3B (Fig. 6). In this respect, the mechanism of BSL’s unwinding with concomitant U2/intron pairing is reminiscent of a toehold-mediated strand invasion reaction^59–61^, where three exposed bases of BSL act as an internal toehold (Fig. 6a, b, d). This type of strand exchange is initiated by a single-stranded oligomer (i.e., the invading strand) binding to an unpaired domain (the “toehold”) of a double-stranded duplex (for a review see ref. ^61^). The toehold can also originate in the loop of a hairpin^60^, as might be the case of BSL (Supplementary Fig. 12).

The transition from the BSL to the precursor U2/intron duplex is likely facilitated by the stepwise action of PRP5/DDX46, TAT-SF1, and SF3A2 (Supplementary Fig. 4)^23,24,62^. Studies in yeast indicate that Prp5p’s (human PRP5/DDX46) ATPase activity is required to displace Cus2p (human TAT-SF1) from SF3B1^62^. Consequently, it has been proposed that Prp5-dependent destabilization of Cus2p results in BSL’s disruption, thereby enabling the extended pairing between U2 and the intron, during prespliceosome’s formation^62^.

TAT-SF1 is present in the cryo-EM structure of the 17S U2 snRNP near the BSL^22^. For structural reasons, displacement of TAT-SF1 is required before the precursor U2/intron duplex can form in the A3′-SSA complex (Fig. 6a, b, d). Indeed, TAT-SF1 is absent from A3′, suggesting that PRP5/DDX46 has already acted (Supplementary Fig. 4b–e). Consistently, while DDX46 is visible in the 17S U2 snRNP, we do not detect its density at the equivalent location of A3′, suggesting relocation or destabilization (Supplementary Fig. 4a). Thus, DDX46 may induce the dissociation of TAT-SF1, which in turn liberates the BSL to create space for the strand exchange (Fig. 6)^22^. Next, SF3A2 would bind the precursor helix mostly via the sugar-phosphate backbone of U2, likely to aid in its formation and stabilization. Importantly, as SF3B1 remains in the open conformation and does not appear to contact RNA, SF3A2 may act as a placeholder to direct the extension of the precursor U2/intron duplex towards the hinged pocket of SF3B1 (Fig. 6b–d).

In the second step—the transition from A3′ to the A complex—the precursor extends with the U2/BS duplex, the bulged BS-A occupies the hinged pocket, SF3B1 clamps on the U2/BS duplex and the PPT binds firmly a deep channel framed by H4–H7 repeats (Fig. 6b, c). The U2/BS duplex extension is likely to precede SF3B1 clamping, given that strong BS can outcompete weaker modulators^32,34^. However, SF3B1’s function may still be required to stabilize the base-pairing interactions between U2’s G31-U34 and four intron nucleotides flanking the BS-A (−1 to +3), especially for “weak”, more degenerate intron substrates^32,34^.

By capturing a snapshot between E and A complexes, the structure of A3′-SSA indicates how introns are recognized and selected stepwise by the U2 snRNP. Our analyses suggest that following the initial U2 snRNP recruitment to the intron, the BSL unwinds via a strand-invasion mechanism to generate the precursor U2/intron duplex. Extension of this duplex enables subsequent selection of the BS-A and SF3B1’s clamping, thereby marking the intron’s commitment for splicing (Fig. 6).

### Structural basis of splicing modulation as a differential inactivation of branch sites

**Fig. 7:**
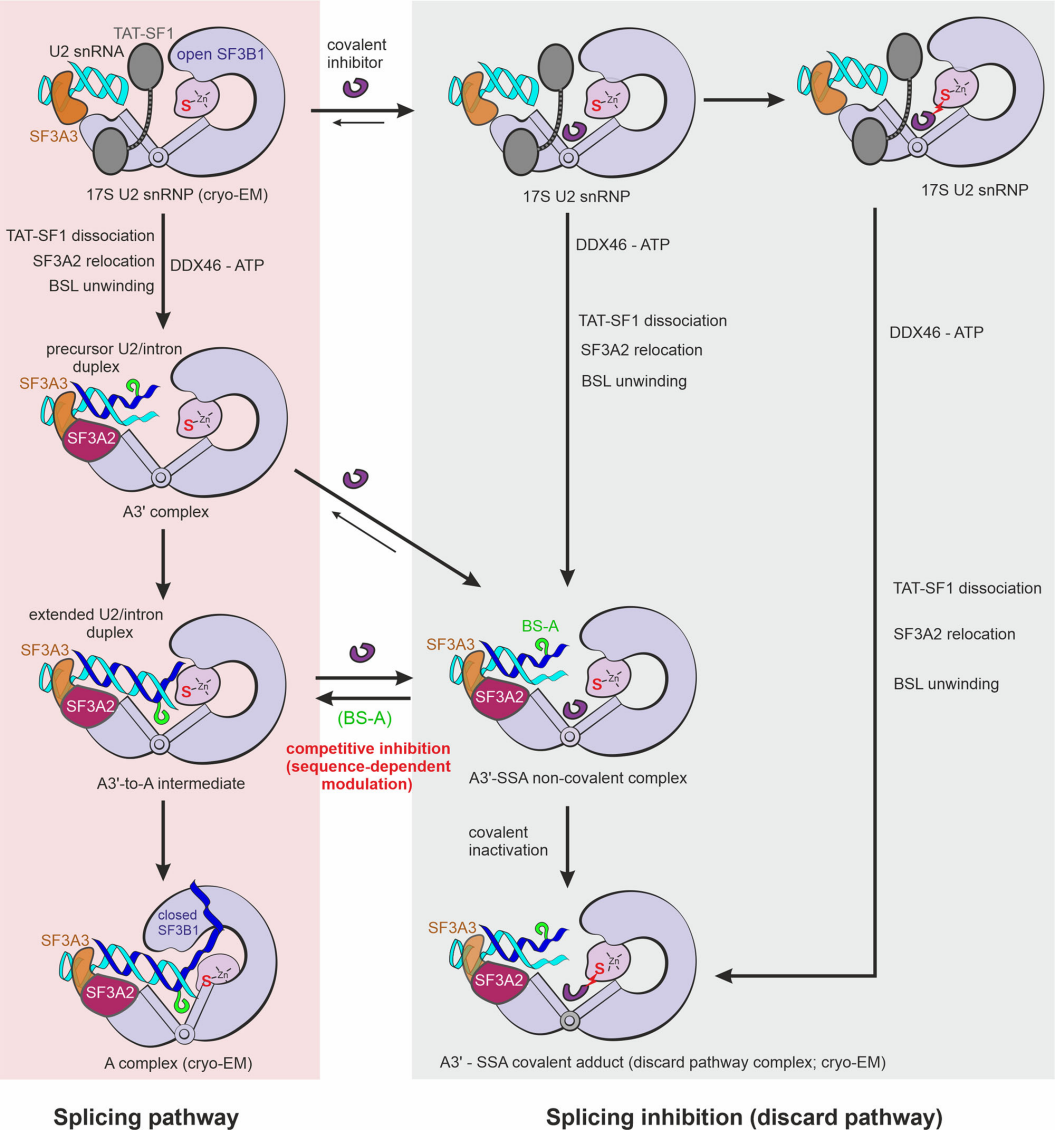
Model of the early splicing pathway under normal and drug-induced conditions. The two pathways are presented in two columns, on distinct backgrounds. Note that modulators can enter the splicing pathway at various stages upon binding the hinged pocket in the open conformation of SF3B1. The competitive inhibition depends on the intron sequence at the BS and occurs before the chemical coupling of covalent inhibitors, thereby leading to differential inhibition of splicing (i.e., modulation). After covalent coupling, the complexes are irreversibly inactivated, resulting in global inhibition of splicing. Except for the indicated covalently inactivated states, the proposed schematic might be largely valid for non-covalent inhibitors as well, including pladienolide derivatives or SSE. The structures determined by cryo-EM are indicated. The SSA-bound 17S U2 snRNP complexes are inferred from the accessibility of the inhibitor’s binding pocket within the cryo-EM structure of the 17S U2 snRNP (PDB 6Y50). The putative transitions between the SSA-bound 17S U2 snRNP and A3′-SSA are inferred from the apparent absence of sterical interference between the bound inhibitor, the intron, and exchanged prespliceosome subunits.

The A3′-SSA structure reveals that FR901464 analogs, such as SSA or SD6, act at the latest stages of intron selection by the U2 snRNP by “locking” the SF3B1 subunit in an open state and preventing the formation of the extended U2/intron duplex. As covalent inhibitors, SSA and SD6 likely bind to and irreversibly modify any complex where SF3B1 exhibits the open conformation, including the isolated SF3B, the 17S U2 snRNP, or A3′-like complexes (Fig. 7). Once the coupling occurs, the covalently modified complexes are likely discarded from the splicing pathway. However, covalent inhibitors can be displaced from the binding pocket before the irreversible coupling occurs (i.e., during the initial binding of inhibitors to the target, when the two form a short-lived reversible intermediate)^63,64^. The competition between BS-A and inhibitors may occur during the conversion of A3′-like to A complexes, explaining why, similar to the pladienolide derivatives, SSA/SD6 elicits differential inhibition of splicing in cells (i.e., splicing modulation; Fig. 7 and Supplementary Fig. 13)^31,32,42^.

The differential response of splicing events to modulators in vivo correlates with the intron’s sequence and length, the conservation/strength of its BS and PPT, as well as the presence of additional motifs upstream of the BS^31^. Sequence-dependent effects were also observed in vitro for less potent SF3B modulators^32,34^. The structure of A3′-SSA shows that SSA’s binding to the hinged pocket allows the initial U2-intron pairing in the form of the precursor duplex while preventing its further extension (Fig. 6). This configuration can explain why degenerate BS motifs are more sensitive in vivo to SSA^43^ or pladienolide derivatives^31,34,35^, as their reduced complementarity to U2 lowers the capacity to outcompete the modulator from the hinged pocket. Furthermore, our model postulates that the efficient extension of the precursor U2/intron duplex is facilitated by PPT-SF3B interactions, rationalizing why weaker/degenerate PPTs increase sensitivity to splicing modulators (Fig. 6b, c)^31^.

Very intriguingly, SSA can induce the U2 snRNA base-pairing upstream of the BS^43^, and the presence of alternative BS motifs upstream of the bonafide BS can lead to drug resistance^31^. Deletion of the BS-A from the upstream motifs eliminates drug resistance, indicating a ligand-induced relocation of the U2 snRNP by an unknown mechanism^31,43^. The strand invasion mechanism, which we propose here, can explain how the U2 snRNP may relocate while maintaining permanent contact with the intron. Thus, when the bonafide BS is too weak/degenerate to outcompete a modulator, the U2 snRNP might slide back in an A3′-like state that continuously exchanges the intron sequence in a “scanning” process (Supplementary Fig. 12c). Once a “stronger BS” motif is detected upstream, the U2/BS helix extension occurs, with the BS-A being extruded from the RNA duplex and able to outcompete the modulator. Finally, the SF3B1 clamps on the U2/intron duplex to “lock” the U2 snRNP in place and signal commitment for splicing from the selected BS. From this perspective, the U2 snRNP would glide along the intron as a pulley device with auto-locking capabilities (Supplementary Fig. 12c). Further investigations are required to identify the molecular basis of this relocation and its potential significance for alternative splicing.

The A3′-SSA structure explains the primary mechanism of modulation as a complementarity-dependent competition between BS-A and the SF3B ligands (Fig. 7). The mechanism might be largely valid for all modulators that bind SF3B, including pladienolide and herboxidiene derivatives. Future comparative studies of prespliceosomes arrested with different compounds and on different introns might shed light on possible additional features that tune splicing modulation.

## Methods

### Engineering, expression, and purification of a minimized SF3B core complex lacking SF3B3’s BPB domain

The first generation of SF3B core constructs encompassed the HEAT domain of SF3B1 (residues 453–1304), PHF5A (residues 1–98), SF3B3 (lacking the internal 1068–1085 residues), and full-length SF3B5, and retained an intact modulator binding pocket^20,34^. This minimal SF3B core packed into orthorhombic, plate-like crystals, which diffracted X-rays anisotropically to a resolution of ~2.9–3.1 Å^34^. As our attempts to crystallize this SF3B core variant in the presence of epoxy modulators (i.e., spliceostatins, sudemycins) or soak crystals with spliceostatins/sudemycins were not successful, we have engineered a second-generation construct lacking the more flexible BPB domain of SF3B3 (residues 442–772). SF3B3’s BPB domain does not establish contacts with the other SF3B subunits^20^ and adopts different poses in various SF3B structures^20,26,27^. By making use of both the multiple cloning and of the Cre recombinase cassettes^34^, we, thus, constructed a single Multibac acceptor plasmid harboring all four SF3B core genes^65^, synthesized initially as codon-optimized genes for expression in insect cells^20^. Like in our previous SF3B constructs^20,34^, we dual-tagged the SF3B3 and the PHF5A subunits of the complex with cleavable 10xHIS and GST (Glutathione *S*-transferase) tags, respectively, and kept similar domain borders, except for SF3B3^34^. In the case of the SF3B3 subunit, in addition to the deletion of the internal 1068–1085 region, we substituted the entire BPB domain with a GGNGNSG linker by round-the-horn PCR mutagenesis (Supplementary Table 4).

In trial expression and protein purification experiments, we observed an apparent tendency of the SF3B^ΔBPB^ core complex to aggregate at concentrations required for crystallization. We adapted our purification procedure (see ref. ^34^ for additional details on the previous purification protocol) by carrying out the final size-exclusion chromatography at a higher salt concentration and substituted the reducing agent for TCEP (Tris (2-carboxyethyl) phosphine). Briefly, Sf9 or High Five insect cells were infected with recombinant baculoviruses and cultured, as previously described^34^. The harvested cells were then gently resuspended with a Dounce homogenizer in the lysis buffer (50 mM HEPES-KOH pH 7.9, 600 mM KCl, 15% (v/v) glycerol, 4 mM DTT), supplemented with the cOmplete cocktail of protease inhibitors (Roche), and lysed by sonication on ice. The crude lysate was subsequently cleared by centrifugation and passed through a 0.45 µM sterile syringe filter before incubation with ~25–30 mL (bed volume) Glutathione Sepharose HP resin (GE Healthcare) for ~2 h at 4–8 °C. The affinity resin was subsequently collected in a gravity-flow column and washed thoroughly with the lysis buffer. The bound SF3B^ΔBPB^ complex was eluted from the affinity resin with the elution buffer (50 mM HEPES-KOH pH 7.9, 500 mM KCl, 10% (v/v) glycerol, 2 mM DTT, 30 mM l-glutathione reduced) and digested with TEV (Tobacco Etch Virus) and HRV (Human Rhinovirus) 3 °C proteases overnight, at 4–8 °C. The salt concentration was then reduced by slow dilution with the dilution buffer (50 mM HEPES-KOH pH 7.9, 25 mM KCl, 20% (v/v) glycerol, 2 mM DTT) and the sample applied onto a 5 mL HiTrap Q Sepharose HP column (GE Healthcare) equilibrated in the buffer A (20 mM HEPES-KOH pH 7.9, 200 mM KCl, 10% (v/v) Glycerol, 1 mM TCEP). The sample was eluted from the column using a linear gradient (0–30%) formed between buffer A and buffer B (20 mM HEPES-KOH pH 7.9, 1 M KCl, 5% (v/v) glycerol, 1 mM TCEP). SF3B^ΔBPB^ peak fractions devoid of nucleic acid contaminants were then concentrated by ultrafiltration using the Amicon Ultra-15 centrifugal filter (50 kDa MWCO, Merck) and further applied to a HiLoad 16/600 Superdex 200 pg column (GE Healthcare) pre-equilibrated in the gel filtration buffer (20 mM HEPES-KOH pH 7.9, 400 mM KCl, 5% (v/v) glycerol, 1 mM TCEP). SF3B^ΔBPB^ peak fractions were then concentrated by ultrafiltration to ~8–10 mg/mL, aliquoted and flash-frozen in liquid nitrogen, and stored at −80 °C. As in the case of our previous recombinant SF3B core, SF3B^ΔBPB^ had an apparent 1:1:1:1 stoichiometry and, as expected, eluted later from the size-exclusion column (with a retention volume of ~11.02 mL vs. ~10.63 mL)^34^. Overall, this indicates that the removal of the BPB domain of SF3B3 did not significantly affect the structural organization and the solution behavior of the remaining subunits or weaken/destabilize the protein–protein interaction interfaces of SF3B^ΔBPB^.

### Crystallization and structure determination of SF3B^ΔBPB^ in complex with spliceostatin/sudemycin and pladienolide modulators

To obtain structures of SF3B^ΔBPB^ in complex with sudemycin/spliceostatin and pladienolide modulators, we have assembled the protein-ligand complexes in vitro by mixing the purified recombinant complex at 4–5 mg/mL final concentration with an excess of the small-molecule compounds. The splicing modulatory compounds used for structural studies (spliceostatin A (SSA), sudemycin D6 (SD6), spliceostatin E (SSE), and pladienolide D (PD)) were synthesized as previously described^33,35,66,67^ and dissolved in DMSO (dimethyl sulfoxide). SSA and PD were added in a ~5-fold molar excess from the 10 mM DMSO stock, whereas the significantly weaker modulators SD6 and SSE were added in a ~10-fold excess over SF3B^ΔBPB^. The samples were then incubated on ice for ~1–12 h, centrifuged, and subjected to extensive crystallization trials. To this end, we explored more than ~2000 commercially available crystallization conditions and different crystallization setups. We have succeeded in further optimizing and collecting data from two new SF3B crystal forms belonging to the orthorhombic (*P*2_1_2_1_2_1_) and trigonal (*P*3_2_21) symmetry groups. Orthorhombic crystals of SF3B^ΔBPB^ in complex with SSA, SD6, SSE, and PD were grown by vapor diffusion at 4 °C in 1 + 1 µL hanging drops equilibrated over a reservoir solution consisting of 0.1 M Bis-Tris propane pH 7.15–7.45, 0.2 M KSCN, 11–12% PEG-3350 (w/v), and 10–15% glycerol (v/v). These crystals were harvested at 4 °C and cryoprotected by stepwise transfer to the reservoir solution supplemented with 25% (v/v) ethylene glycol before flash-freezing in liquid nitrogen. Trigonal crystals of SF3B^ΔBPB^ in complex with SSA and SD6 were obtained at 4 °C in 1 + 1 µL hanging drops placed over a reservoir solution containing 0.1 M HEPES pH 7.17–7.42, 0.2 M MgCl_2_, 25–27.5% (v/v) PEG-400. Before being snap-cooled in liquid nitrogen, the trigonal SF3B^ΔBPB^ crystals were sequentially transferred to the corresponding reservoir solution containing 40% (v/v) PEG-400. Diffraction data were collected from single crystals at 100 K on the PILATUS 6M-F or EIGER2 16M detectors (Supplementary Tables 2 and 3), indexed, integrated, and scaled with XDS^68^, and further merged in AIMLESS^69^. The trigonal crystals of SF3B^ΔBPB^ diffracted X-rays up to ~2.9–3.0 Å, as our previous SF3B core crystals^34^. Importantly, diffraction data collected from our best orthorhombic crystals extended to a resolution of ~2.3 Å. To our knowledge, this represents the highest resolution structural data available for the core of the human SF3B complex. The co-crystal structures of SF3B^ΔBPB^ in the two different space-groups were phased by molecular replacement with Phaser^70^ using a search model lacking the BPB domain of SF3B3. The models were then iteratively rebuilt in Coot^71^ and refined with phenix.refine^72^. Data collection and refinement statistics are provided in Supplementary Tables 2 and 3. Geometric restraints for the refinement of spliceostatin/sudemycin and pladienolide modulators were generated with Grade (Global Phasing Limited) and edited in phenix.reel^73^. The SF3B modulators were located and modeled largely based on the residual *mFo-DFc* and polder omit maps. In addition, anomalous difference maps, calculated from diffraction data collected at the Zn K-edge (Supplementary Tables 2 and 3), as well as composite omit maps^74^ were used to accurately rebuild the Znf1 motif of PHF5A (Supplementary Fig. 7b). Unexpectedly, the initial refinement of the SF3B^ΔBPB^ structures in complex with SSA and SD6 (i.e., epoxy modulators) revealed a strong density element between the C26 residue of the PHF5A subunit and the epoxy-tetrahydropyran moiety of the small-molecule compounds. We interpreted the continuous positive density as an intermolecular covalent bond resulting from the reaction of C26 and with the epoxy group of SSA/SD6. We located the remaining tetrahydropyran/cyclohexane group and the aliphatic side-chain of the epoxy modulators in the BS-A binding pocket of SF3B, whereas the diene group is more exposed to the solvent (Supplementary Fig. 6). The terminal acetate group of SSA has weaker density and higher temperature factors compared to its neighboring groups, consistent with it being likely more susceptible to hydrolysis during crystallization and/or radiation damage. Pladienolide D (PD) was modeled and refined as detailed in our previous work^34^. Structural analyses were carried out in PyMOL version 2.3.3 (Schrodinger LLC) and the protein-ligand interactions were calculated with Arpeggio^75^. For crystallization, we used the same batch of SSA and SD6 characterized by Teng and co-workers^33^.

### Cloning and in vitro transcription of the A3′-exon pre-mRNA substrate

The A3′−5′SS exon construct was synthesized as a gBLOCK (IDT, Supplementary Table 4) and cloned into a LIC compatible vector (438-C, Dr. Scott Gradia). A *Xba*I restriction site, downstream of the MS2 aptamer sequences, was used to linearize the plasmid and generate in vitro transcription templates. The final construct includes 54 nts of the MINX intron, upstream of the 3′SS, the second MINX exon, a “strong” 5′SS, followed by three consecutive MS2 aptamer sequences:

5′-GGGCGCAGUAGUCCAGGGUUUCCUUGAUGAUGUCAUACUUAUCCUGUCCCUUUUUUUUCCACAGCUCGCGGUUGAGGACAAACUCUUCGCGGUCUUUCCACAGGUAAGUUGGAAGCAUGUAGAACCUUGGAUCCGAUAUCCGUACACCAUCAGGGUACGAGCUAGCCCAUGGCGUACACCAUCAGGGUACGACUAGUAGAUCUCGUACACCAUCAGGGUACGGAAUUCU-3′

MS2-tagged RNA substrates used for the assembly of A3′−5′SS exon complexes were prepared by T7 run-off transcription of the corresponding linearized DNA templates (i.e., obtained by digestion with *Xba*I). The in vitro transcription reactions were set up and carried out as previously described^17^, except omitting the m^7^GpppG cap analog. The in vitro transcribed RNA was recovered using the MEGAclear kit (ThermoFisher Scientific) following the manufacturer’s protocol. The quality of the transcribed RNA templates was assessed on a denaturing PAGE gel stained with SYBR Gold, largely as described in ref. ^17^.

### Assembly and purification of the A3′ complex arrested in the presence of SSA

HeLa S3 cells were cultured in a bioreactor^17^ and the HeLa nuclear extract was prepared as previously described, aliquoted, and stored in Roeder C buffer at −80 °C^17^. The A3′-exon complex was allowed to form in vitro in the HeLa nuclear extract upon the addition of MS2-tagged pre-mRNA substrates, while the progression of the splicing reaction was prevented by spliceostatin A (SSA).

Both trial and large-scale splicing reactions were set up on ice largely as previously described^17^. A typical splicing reaction contained 20 mM HEPES-KOH pH 7.9, 3 mM MgCl_2_, 2 mM ATP, 20 mM CP (Creatine Phosphate), 20% (v/v) HeLa nuclear extract (in Roeder C buffer) and 10 nM pre-mRNA substrate. SSA was added to the nuclear extract prior to the initiation of the splicing reaction to 500 nM final concentration, followed by incubation of the extract for 1 h on ice. In parallel, the in vitro transcribed pre-mRNA substrate was mixed with a ~20-fold molar excess of recombinant MBP-MS2, followed by incubation for 1 h on ice, as described in ref. ^17^. Prior to the start of the reaction, the pre-mRNA substrate (10 nM final concentration) complexed with MBP-MS2 was added to the splicing mix and the splicing reaction was transferred immediately to a water bath equilibrated at 30 °C and incubated for 15′. The splicing reaction was then placed on ice for 5′ and centrifuged at 4 °C for 20 min at 4000 rpm (2890×*g*). The supernatant was carefully decanted to a pre-chilled glass cylinder and loaded on a 5 mL MBPtrap column (GE Healthcare) at 0.5 mL/min equilibrated in the binding buffer (20 mM HEPES-KOH pH 7.9, 75 mM KCl, 1.5 mM MgCl_2_, 5% (v/v) glycerol, 1 mM DTT). The column was then washed extensively with the binding buffer and then with the washing buffer (20 mM HEPES-KOH pH 7.9, 75 mM KCl, 1.5 mM MgCl_2_, 1 mM DTT). The A3′ exon complexes were eluted off the affinity column with the elution buffer (20 mM HEPES-KOH pH 7.9, 75 mM KCl, 1.5 mM MgCl_2_, 2 mM l-maltose). The A3′-exon affinity fractions were loaded separately onto a 14 mL linear 5–20% (w/v) sucrose gradient prepared in G-75 buffer (20 mM HEPES-KOH pH 7.9, 75 mM KCl, 1.5 mM MgCl_2_) and ultracentrifuged in a TST41.14 (Kontron) rotor at 22,000 rpm (~85,852×g) for 13 h 30′ at 4 °C. The sucrose gradients were harvested manually from top to bottom in 0.5 mL fractions or bottom to top in 0.68 mL fractions and analyzed on a denaturing PAGE gel, followed by staining with SYBR Gold and Coomassie (Supplementary Fig. 1b). We used the previously described methods for mass-spectrometry sample preparation, digestion, measurement, and analysis^17,27^ (see also Supplementary Data 1).

### Cryo-EM sample preparation

To account for the relative lability of the A3′-SSA complex (i.e., compared to the later B, C, or P stage complexes), we reconstituted the early pre-spliceosomes under low salt conditions (50–75 mM KCl) and reduced the number of purification steps prior to cryo-EM sample preparation. In addition, a chemical crosslinking strategy was employed to stabilize the more labile samples prior to freezing them in vitreous ice.

The A3′-SSA complex was prepared in a similar manner as for mass-spectrometry analysis, except that the sample was subjected to GraFix after the amylose affinity step. That is, after the amylose affinity selection step, used to capture the assembled complex from the splicing reaction, individual fractions of the sample were applied to a 14 mL 5–20% (v/v) sucrose gradient in the presence of 0–0.1% glutaraldehyde and subjected to ultracentrifugation, as described^76^. In the next step, the sucrose gradient was harvested, and the remaining crosslinking agent was quenched with 50 mM l-aspartate^76^. A3′-SSA fractions containing the U1 and U2 snRNP in an apparent equimolar amount were gently rebuffered to G-75 buffer by ultrafiltration, concentrated to ~0.24–0.26 mg/mL, and then used directly for cryo-EM grid preparation. Although we screened different types of cryo-EM grids (e.g., R2/2, R1.2/1.3, C3.5/1) and grid freezing setups, a compromise between the ice thickness, density, distribution of particles, and image contrast was observed when the A3′-SSA sample was frozen in pure ice on UltrAufoil R1.2/1.3 gold grids (Plano). In this case, the A3′-SSA complex was frozen in vitreous ice by applying ~2.2 µL crosslinked sample to both sides of a glow-discharged grid mounted in a Vitrobot Mark IV (ThermoFisher Scientific) which was operated at 4 °C and 100% humidity. The excess sample was blotted away for 2 s using a blot force of 7 and the grids were snap-frozen in liquid ethane cooled by liquid nitrogen.

### Cryo-EM data collection and image processing

Cryo-EM images of the A3′-SSA complex were collected using SerialEM^77^ on a high-end Titan Krios transmission electron microscope (ThermoFisher Scientific) operated at 300 kV in EFTEM mode and equipped with a BioQuantum GIF energy filter (slit width 20 eV). We acquired 10,494 “good” movie stacks of the A3′-SSA complex from the same grid in two separate sessions. The cryo-EM micrographs were recorded on a K3 detector (Gatan) in counting mode at a magnification of 81,000× corresponding to a pixel size of 1.05 Å at specimen level (see also Supplementary Fig. 1 and Supplementary Table 1). Each of the A3′-SSA micrographs was acquired over an exposure time of ~2 s and was dose-fractionated into 40 frames, resulting in a total dose of ~41.41 e^−^/Å^2^ and a dose rate of ~1.04 e^−^/Å^2^/frame.

The raw cryo-EM movie stacks were preprocessed on-the-fly in Warp (i.e., motion correction, dose-weighting, and CTF estimation) and particles were picked using a custom BoxNet neural net^78^, which was separately retrained against each of the two A3′-SSA data sets. To account for the elongated nature of the complex and accommodate its different conformational states, we extracted the A3′-SSA particle images in a large 680 /680 px (714/714 Å) box. Data processing was largely performed in RELION-3.0 or RELION-3.1-beta^79^ and cryoSPARC v2^80^.

The two data sets of the A3′-SSA particle images (in total, ~1.05 mln “particles”) were initially binned 2× and subjected separately to 2D classification, followed by supervised 3D classification in cryoSPARC. Next, the cleaned subset of particles (351,004 particles) was combined and re-extracted in RELION and further classified in 2D. Importantly, 2D class averages of A3′-SSA show clear secondary structure features for the more rigid U2 5′ module of the complex (Supplementary Fig. 1). The U2 3′ module of the U2 snRNP appears dynamic and its density is blurred in 2D, as it was also recently observed for the isolated U2 17S particle^22^. An additional peripheric density element is located on the opposing side from U2 3′ module. This density is less defined and, likely, contains other proteins detected in the proteome of the A3′-SSA complex. The subset of “good” particle images (i.e., displaying secondary structure features) resulting from 2D classification (164,107 particles) was further refined in RELION to obtain a reference map for the A3′-SSA, which was then low-passed to 60 Å and applied to all subsequent 3D refinement and classification steps. To potentially include less abundant views of the A3′-SSA, we went back to our initial 2× binned particle sets and performed 3D classification in RELION with 8 classes and the low-passed A3′-SSA reference volume obtained in the previous step. Particles representing the A3′-SSA complex were merged and then subjected to another round of 3D classification with image alignment and a soft mask applied on the U2 5′ module of the complex. The resulting subset of 111,972 particles was refined in 3D to obtain a ~8.4 Å overall map of the A3′-SSA complex, as estimated using the gold-standard criterion of FSC (Fourier shell correlation) = 0.143 in RELION (Supplementary Fig. S1E). The quality of the overall reconstruction (map M1) was limited by the intrinsic structural flexibility of the complex and, likely, the biased orientation of particles in ice. To improve the local density of the individual modules of the complex, we resorted to focused classification and refinement with soft solvent masks applied to the U2 3′ and U2 5′ modules. After re-extracting, re-centering, and refining the particles at 1.05 Å/px in a 480 px box, we performed an additional 3D classification without image alignment with 6 classes and a soft mask applied on the U2 5′ region. This classification approach led to a more conformational homogenous subset of particle images (78,262 particles) which was further subjected to iterative CTF refinement (per-particle defocus, per-micrograph astigmatism) and Bayesian polishing in RELION-3.1-beta^79^. The resulting ~3.1 Å map of the U2 5′ module (Supplementary Fig. 2, map M2) allowed us to model a large part of the SF3B and SF3A subcomplexes, the precursor U2/intron duplex, and to locate the SSA inhibitor bound to an endogenous spliceosome complex. Refinement of the same subset of particles with a “loose” mask resulted in map M3 of the U2-5′ module, which also includes the “top region” of the complex. 3D classification with a soft mask applied to the U2 core and further refinement led to a map (map M4) with improved features for the U2 3′ domain (Supplementary Fig. 2) which allowed us to further extend our model.

### Cryo-EM model building and refinement

To enable model building, the U2 5′ module map was automatically sharpened in RELION by applying a negative B factor of approximately −54 Å^2^. In addition, local map sharpening with LocScale^81^, as implemented in CCP-EM, and phenix.auto_sharpen^82^ provided alternative maps for interpreting and modeling of the more peripheric, low-resolution regions of the complex. Global and local resolution of the A3′-SSA maps were estimated in RELION (Supplementary Fig. 1).

We built an initial model for the U2-5′ module of A3′-SSA, first by rigid-body fitting the ~2.3 Å crystal structure of the SF3B^ΔBPB^-SSA (Fig. 2 and Supplementary Fig. 5). The more peripheric SF3B4 (residues 12–89, RRM1) and SF3B2 (458–532, 566–666, 680–687 residues) subunits we modeled based on the cryo-EM structures of the human pre-B and B^act^ complexes^27,47^, whereas the BPB domain of SF3B3 was built based on our previous structure of the SF3B core complex^20^ (PDB 5IFE). The SF3A2 (residues 42–85, zinc-finger domain) and SF3A3 (residues 392–482, zinc-finger domain) subunits were docked separately with their initial poses obtained from the available models of the human B^act^ complex^27^ (PDB 6FF4/6FF7). U2 snRNA and the U2/intron precursor duplex were modeled based on the human B^act^ complex (PDB 6FF4/6FF7) and manually adjusted to fit the cryo-EM density in Coot and ISOLDE. The model of the U2-5′ module was refined with phenix.real_space_refine using manually curated base-pairing and stacking restraints, rebuilt, and further extended in Coot. The U2 3′ model was obtained from the human pre-B complex^47^ and fitted as a rigid body in Chimera in the corresponding map (map M4).

### Mass spectrometry and data analysis

The SF3b complex (1 μM) was incubated in 20 mM HEPES pH 8.0, 200 mM KCl, 5% glycerol, and 1 mM TCEP with a 2-fold excess of compound (2 μM) at 4 °C overnight. Mass analyses were carried out on a Thermo Scientific Q-Exactive HRM (ESI source, 3.5 kV ionization voltage, 300 °C capillary temp., 55 arb sheath gas, aux gas flow rate at 5 L/min, S-lens RF level 50) coupled with Accela Open AS 1250. Samples (5 µL) were desalted on a C18 column (Thermo Scientific Accucore 2.1 × 150 mm, 2.6 µm) for 5 min prior to gradient run. Gradient started from 20 to 65% eluent B for 5 min. Eluent A consisted of 0.1% formic acid in water and eluent B consisted of 0.1% formic acid in acetonitrile. The flow was set to 400 µL/min. All solvents were LC/MS grade (Thermo Scientific). The mass spectrometer was run in a positive mode collecting full scan at *R* = 70,000 from *m*/*z* 400 to *m*/*z* 2000. Data collected with Xcalibur 3.1 software.

Xcalibur raw files were processed using BioPharma Finder 2.0 (Thermo Scientific) with Xtract deconvolution algorithm. Peak averaged over selected retention time to generate source spectra from TIC chromatogram trace, chromatogram parameters set to *m*/*z* 400 to 2000. Outputs from the deconvolution algorithm include a mass range from 10,000 to 160,000 with a mass tolerance of 20 ppm and a charge state range from 10 to 100. The target mass is the estimated mass of protein or protein + compounds with a noise rejection of 95% confidence.

### Cell viability assays

HCT116 PHF5A-C26H, HCT116 PHF5A-K29A, HCT116 PHF5A-K29R, HCT116 EV (empty vector) cell lines were generated by lentiviral overexpression as described^33^. The overexpression of PHF5A in blasticidin-selected cells was confirmed by western blotting. For CellTiter-Glo analysis, 2000 cells (100 µL) were seeded in each well of a 96-well plate. The next day, cells were treated with compounds at 10-point three-fold serial dilution starting with a top dose of 10 µM. 72 h post compound addition, CellTiter-Glo reagent (100 μL) was added to the cell medium, incubated, and the signal was measured on EnVision Reader (PerkinElmer). The luminescence value from each treatment sample was normalized to the average value of the respective DMSO control. The dosage response curve plots were generated using Prism 8 (GraphPad) and fit using non-linear regression analysis.

### Scintillation proximity assays for competitive binding

SF3B core complexes (wild-type and PHF5A-C26H) used for SPA were purified as described before^34^. For SPA, the anti-FLAG antibody (Sigma-Aldrich) was immobilized to anti-mouse PVT SPA beads (PerkinElmer) as described^34^. 100 µL binding reactions were prepared with 50 µL bead slurry and 25 nM purified SF3B core complex in buffer (20 mM HEPES pH 8, 200 mM KCl, 5% glycerol), and different compounds in a 10-point three-fold serial dilution with the top concentration of 4 µM were used. After the mixture was pre-incubated for 30 min, 10 nM [^3^H]-probe ([^3^H]-labeled pladienolide B) was added to the mixture and incubated for 30 min, and luminescence signals were read using a MicroBeta2 Plate Counter (PerkinElmer). Prism 8 (GraphPad) was used for the non-linear regression curve fitting of the data.

### Reporting summary

Further information on research design is available in the Nature Research Reporting Summary linked to this article.

## Supplementary information

Supplementary Information

Supplementary Dataset 1

Reporting Summary

## Data Availability

Coordinates and structure factors were deposited in the Protein Data Bank (PDB accession codes: 7B9C, 7B0I, 7B91, 7B92, 7OMF, 7OPI). The cryo-EM maps and the associated coordinates were deposited in the Electron Microscopy Data Bank (EMDB accession code: EMD-12994) and the Protein Data Bank (PDB accession code: 7ONB). Requests for samples, materials, and data should be addressed to vlad.pena@icr.ac.uk. Source data are provided with this paper.

## Source data

Source Data
