# Supplementary material for: Structural basis of intron selection by U2 snRNP in the presence of covalent inhibitors
Source: Nat Commun. 2021 Jul 23;12:4491. doi: 10.1038/s41467-021-24741-1 (PMC8302644; doi:10.1038/s41467-021-24741-1)
Supplement: Supplementary file 1 — Supplementary Information [file 41467_2021_24741_MOESM1_ESM.pdf]

Structural basis of intron selection by U2 snRNP in the presence of covalent inhibitors

Constantin Cretu[1†], Patricia Gee[2], Xiang Liu[2], Anant Agrawal[2], Tuong-Vi Nguyen[2], Arun K. Ghosh[3], Andrew Cook[2], Melissa Jurica[4], Nicholas A. Larsen[2], Vladimir Pena[1*]

[1]Research Group Mechanisms and Regulation of Splicing, The Institute of Cancer Research, Fulham Road 237, SW3 6JB London, United Kingdom

[2]H3 Biomedicine, Inc., Cambridge, MA 03129, USA

[3]Departments of Chemistry and Medicinal Chemistry, Purdue University, West Lafayette, Indiana, 47907, USA

[4]Department of Molecular, Cell and Developmental Biology, University of California, Santa Cruz CA95064, USA

[†]Present address: Cluster of Excellence Multiscale Bioimaging (*MBExC*), Universitätsmedizin Göttingen, Robert-Koch-Str. 40, 37075 Göttingen, Germany

Lead Contact: Vladimir Pena

[*] Correspondence to vlad.pena@icr.ac.uk

Supplementary Figures

a

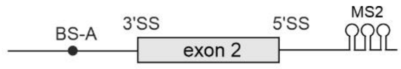

c

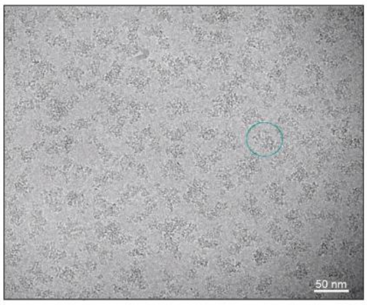

b

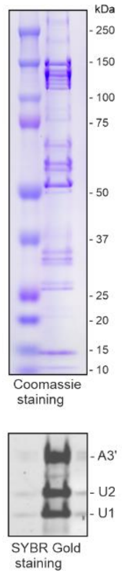

	Protein	Alternative name	Uniprot	MW	Peptides	Spectra
**17S U2 snRNP**	SF3B1	SF3b155	O75533	146 kDa	83	389
	SF3B2	SF3b145	Q13435	100 kDa	47	166
	SF3B3	SF3b130	Q15393	136 kDa	69	442
	SF3B4	SF3b49	Q15427	44 kDa	7	30
	SF3B5	SF3b10	Q9BWJ5	10 kDa	4	6
	SF3B6	SF3a14a, p14	Q9Y3B4	15 kDa	7	50
	PHF5A	SF3b14b	Q7RTV0	12 kDa	6	26
	SF3A1	SF3a120	Q15459	89 kDa	49	233
	SF3A2	SF3a66	Q05DF2	51 kDa	11	31
	SF3A3	SF3a60	B3KY12	59 kDa	24	92
	SNRPA1	U2-A'	P09661	28 kDa	25	130
	SNRPB2	U2-B"	P08579	25 kDa	10	42
**Sm ring**	SNRPB	Sm-B/B'	B4DVS0	24 kDa	12	95
	SNRPD3	Sm-D3	P62318	14 kDa	9	70
	SNRPD2	Sm-D2	P62316	14 kDa	12	67
	SNRPD1	Sm-D1	P62314	13 kDa	7	54
	SNRPE	Sm-E	P62304	11 kDa	5	22
	SNRPG	Sm-G	P62308	8 kDa	4	16
	SNRPF	Sm-F	P62306	10 kDa	3	8
**17S U2 snRNP related**	DDX46	hPRP5	Q7L014	117 kDa	77	354
	DDX42	SF3b125	Q86XP3	103 kDa	9	9
	DHX15	hPRP43	O43143	91 kDa	37	138
	HTATSF1	Tat-SF1	O43719	86 kDa	4	4
	U2SURP	SR140	O15042	118 kDa	1	1
	PUF60	FIR	Q9UHX1	57 kDa	26	155
	U2AF2	U2AF65	P26368	54 kDa	22	72
	U2AF1	U2AF35	P0DN76	28 kDa	9	26
**U1 snRNP**	SNRPA	U1-A	B2R802	31 kDa	15	78
	SNRNP70	U1-70K	P08621	52 kDa	19	73
	SNRPC	U1-C	P09234	17 kDa	3	5
**U1 snRNP related**	PRPF40A	FBP11, HIP-10	O75400	109 kDa	31	185
	RBM25	RED120	P49756	100 kDa	43	150
	LUC7L3		O95232	51 kDa	16	64
	LUC7L		Q9NQ29	44 kDa	7	13
	LUC7L2		Q9Y383	47 kDa	3	4
**E/A complex factors**	RBM5		P52756	92 kDa	13	21
	SF1	mBBP, ZNF162	Q15637	62 kDa	17	116
	SF4	SUGP1	A5PLN4	73 kDa	6	7
	RBM39	CAPERalpha	Q14498	59 kDa	29	145

d

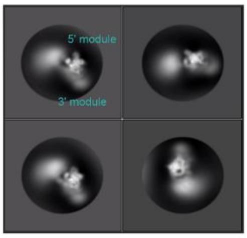

e

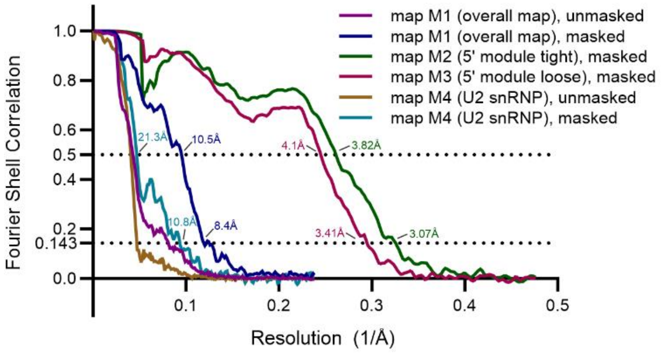

f

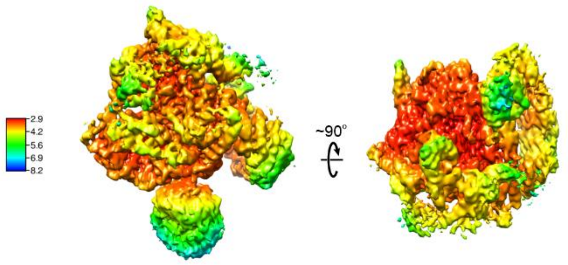

g

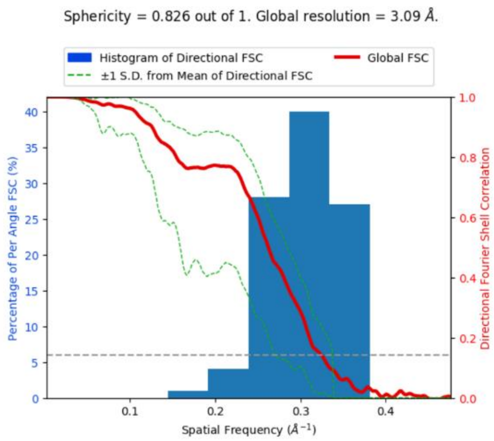

h

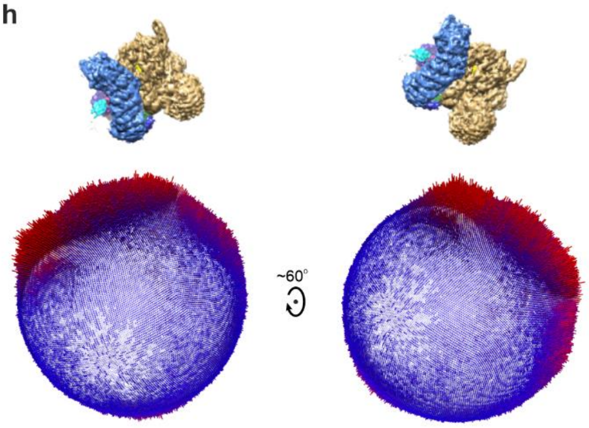

Supplementary Figure 1. Purification, characterization, and cryo-EM structure determination of the A3'-SSA complex. a Schematic of the RNA construct used for the assembly of the human A3' complex. **b** SDS-PAGE analysis of the purified A3'-SSA complex. The protein composition of the A3'-SSA spliceosomes as revealed by LC/MS-MS is shown in the table. The proteins present in the cryo-EM model are highlighted. See also Supplementary Data 1 for a complete overview of the detected peptides. The A3'-SSA complex preparation for cryo-EM analysis was repeated at least three times with similar results. Source data are provided as a Source Data File. **c** Representative cryo-EM micrograph of the human A3'-SSA complex (cropped). The micrograph was visualized with XMIPP3 with the default settings. Two independent cryo-EM datasets of the A3'-SSA were acquired, resulting in comparable 2D classes and 3D reconstructions. However, only the A3'-SSA dataset recorded on the K3 detector is reported in the current manuscript. **d** Typical reference-free 2D class averages of the A3'-SSA complex. **e** Global resolution estimation of the A3'-SSA cryo-EM maps by Fourier Shell Correlation (FSC). The FSC was calculated between two independently refined half maps in RELION. The gold-standard FSC=0.143 criterion indicates a global resolution of ~3.07 Å for the U2 5' module of the complex. **f** Local resolution of the U2 5' module of the A3'-SSA complex. The U2 5' domain map shows a resolution range between ~2.9 Å at the core and ~8.2 Å at its periphery. Local resolution was estimated in RELION and visualized in UCSF Chimera. **g** Directional resolution of the U2 5' module map. The 3D FSC analysis was carried out with the 3D FSC server (3dfsc.salk.edu). **h** Angular distribution plot of the particle images contributing to the focused map of the U2 5' module. The red color indicates a higher number of particles at a given projection angle. The final map of the U2 5' module (map M2, unsharpened) is displayed above the Euler angle distribution plot and is color-coded as in Figure 1.

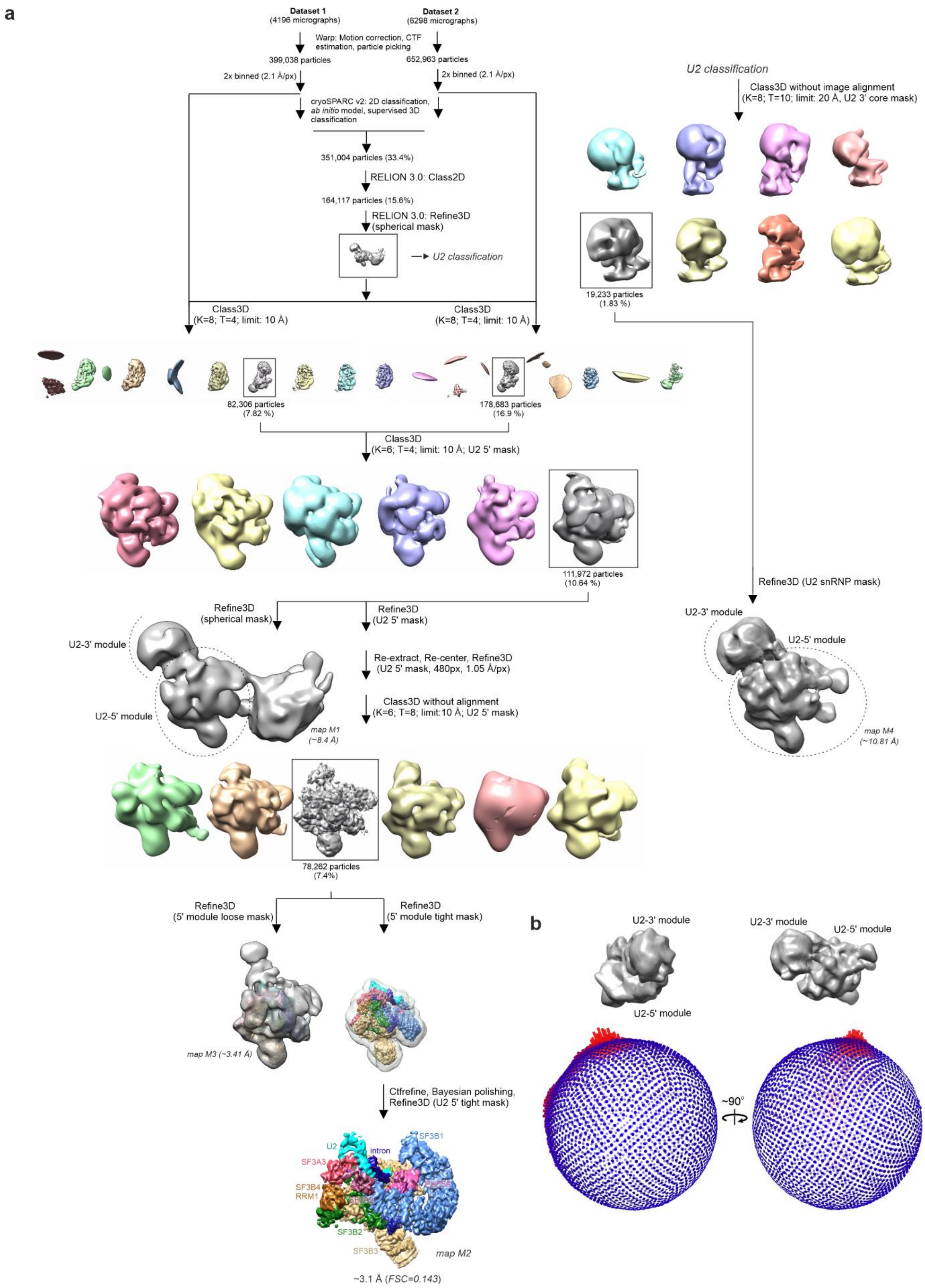
a
Dataset 1
(4196 micrographs)
Dataset 2
(6298 micrographs)
Warp: Motion correction, CTF estimation, particle picking
399,038 particles
652,963 particles
2x binned (2.1 Å/px)
2x binned (2.1 Å/px)
cryoSPARC v2: 2D classification, ab initio model, supervised 3D classification
351,004 particles (33.4%)
RELION 3.0: Class2D
164,117 particles (15.6%)
RELION 3.0: Refine3D
(spherical mask)
U2 classification
Class3D
(K=8; T=4; limit: 10 Å)
Class3D
(K=8; T=4; limit: 10 Å)
82,306 particles
(7.82 %)
178,683 particles
(16.9 %)
Class3D
(K=6; T=4; limit: 10 Å; U2 5' mask)
111,972 particles
(10.64 %)
Refine3D
(spherical mask)
Refine3D
(U2 5' mask)
U2-3' module
U2-5' module
map M1
(~8.4 Å)
Re-extract, Re-center, Refine3D
(U2 5' mask, 480px, 1.05 Å/px)
Class3D without alignment
(K=6; T=8; limit:10 Å; U2 5' mask)
78,262 particles
(7.4%)
Refine3D
(5' module loose mask)
Refine3D
(5' module tight mask)
map M3 (~3.41 Å)
Ctfrefine, Bayesian polishing,
Refine3D (U2 5' tight mask)
U2
intron
SF3B1
SF3A3
SF3B4
RRM1
SF3B2
SF3B3
map M2
~3.1 Å (FSC=0.143)
U2 classification
Class3D without image alignment
(K=8; T=10; limit: 20 Å, U2 3' core mask)
19,233 particles
(1.83 %)
Refine3D (U2 snRNP mask)
U2-3' module
U2-5' module
map M4
(~10.81 Å)
b
U2-3' module
U2-5' module
U2-3' module
U2-5' module
~90°

Supplementary Figure 2. Cryo-EM image-processing workflow of the A3'-SSA complex. **a** Cryo-EM data processing schematic showing the most significant steps of the computational analysis. The detailed image-processing procedure is described in the Methods section. **b** Angular distribution plot of the particle images, which contributed to the cryo-EM map of the U2 snRNP region of the complex.

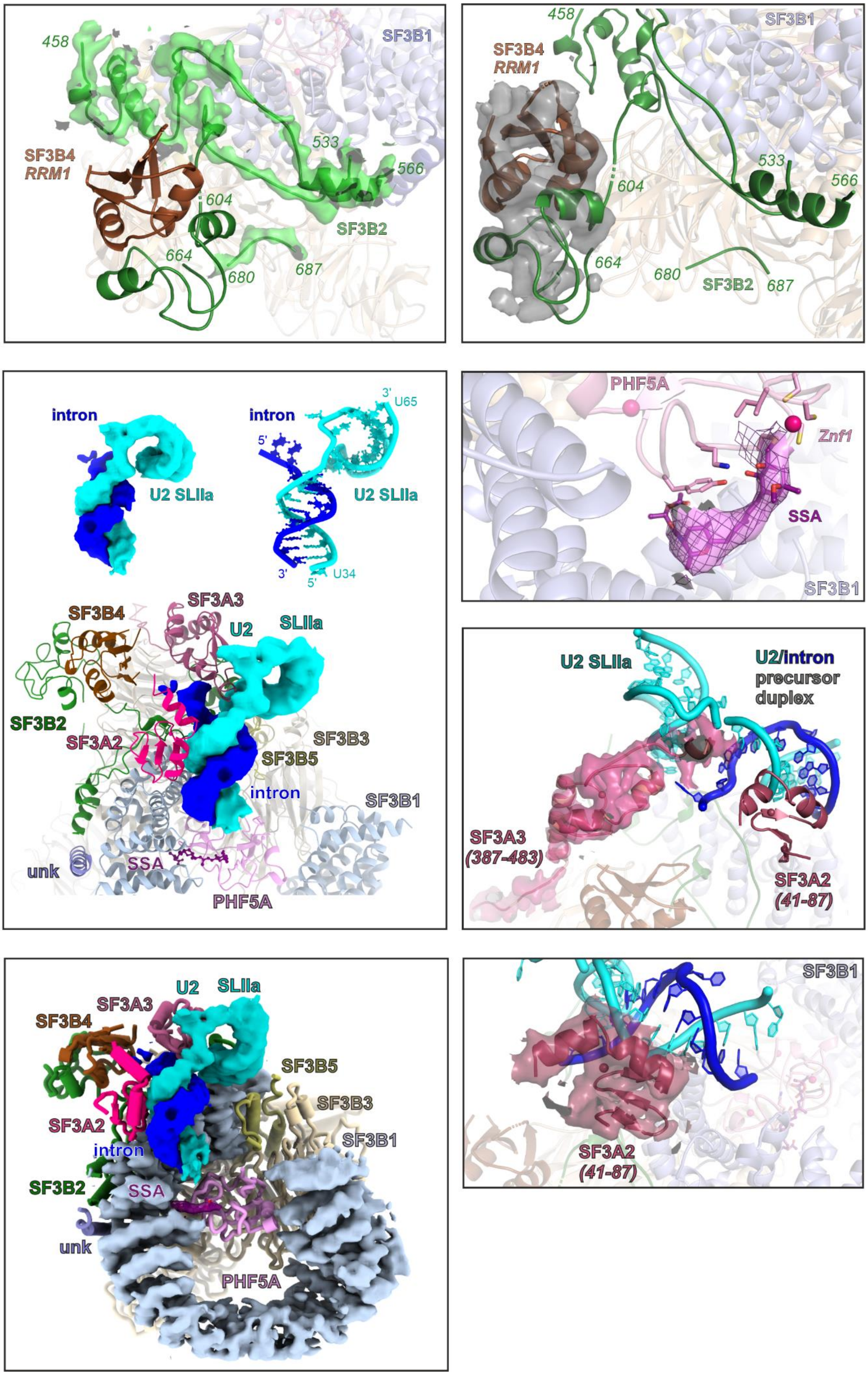

458
SF3B1
SF3B4
RRM1
533
566
604
SF3B2
664
680
687
458
SF3B1
SF3B4
RRM1
604
533
566
664
680
SF3B2
687
intron
U2 SLIIa
intron
3'
U65
5'
U2 SLIIa
3'
5'
U34
SF3B4
SF3A3
U2
SLIIa
SF3B2
SF3A2
SF3B3
SF3B5
intron
SF3B1
unk
SSA
PHF5A
PHF5A
Znf1
SSA
SF3B1
U2 SLIIa
U2/intron
precursor
duplex
SF3A3
(387-483)
SF3A2
(41-87)
SF3B1
SF3A2
(41-87)
U2
SLIIa
SF3A3
SF3B4
SF3B5
SF3B3
SF3A2
SF3B1
intron
SF3B2
SSA
unk
PHF5A

Supplementary Figure 3. Cryo-EM density snapshots of selected regions of the U2 5' module. The subunits of the complex are colored and labeled as in Figure 1.

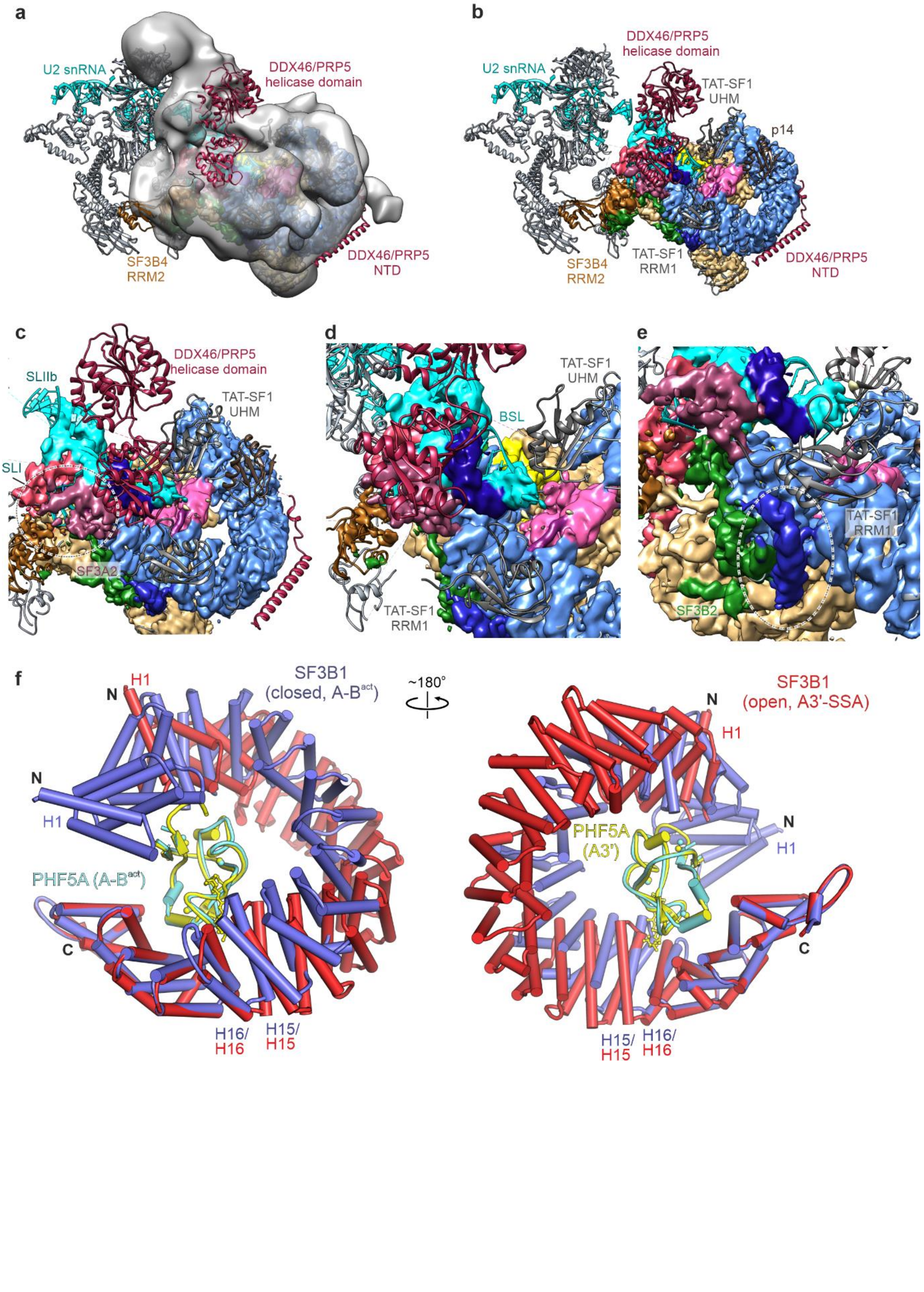
a
U2 snRNA
DDX46/PRP5
helicase domain
SF3B4
RRM2
DDX46/PRP5
NTD
b
DDX46/PRP5
helicase domain
U2 snRNA
TAT-SF1
UHM
p14
SF3B4
RRM2
TAT-SF1
RRM1
DDX46/PRP5
NTD
c
DDX46/PRP5
helicase domain
SLIIb
TAT-SF1
UHM
SLI
SF3A2
d
TAT-SF1
UHM
BSL
TAT-SF1
RRM1
e
TAT-SF1
RRM1
SF3B2
f
SF3B1
(closed, A-B^act)
~180°
SF3B1
(open, A3'-SSA)
N
H1
N
H1
PHF5A (A-B^act)
C
H16/
H16
H15/
H15
N
H1
PHF5A
(A3')
N
H1
C
H15/
H15
H16/
H16

Supplementary Figure 4. Comparison between the 17S U2 snRNP particle and the A3'-SSA complex. a-b Superposition of the 17S U2 snRNP model (PDB 6Y50) onto the U2 5' module of A3'-SSA. A more loosely masked density map (map M3, low-passed to 15 Å) is depicted together with the higher resolution U2 5' map (map M2), colored according to its constituent subunits. Compared to the 17S U2 snRNP structure, DDX46/hPrp5 helicase region is no longer present as its location in the A3'-SSA complex, and the density for its NTD region is absent. **c-d** Remodelling of the 5' end of the U2 snRNA upon the formation of the U2/intron precursor duplex. The U2 SLI, as seen in the 17S U2 snRNP, clashes severely with the zinc finger domain SF3A2 in A3'-SSA, which apparently stabilizes the U2/intron precursor duplex. **e** An unassigned helical element is observed in the A3'-SSA structure at the hinge region of SF3B1. **f** Structural superposition between the SF3B modules from A3'-SSA and B^{act} (PDB 5Z58) complexes, indicating the difference between the open and closed conformations of SF3B1 (red and blue, respectively). Only SF3B1 and PHF5A are shown for clarity's sake. Note that PHF5A and the HEAT repeats H16-H20 are very similar in the two structures.

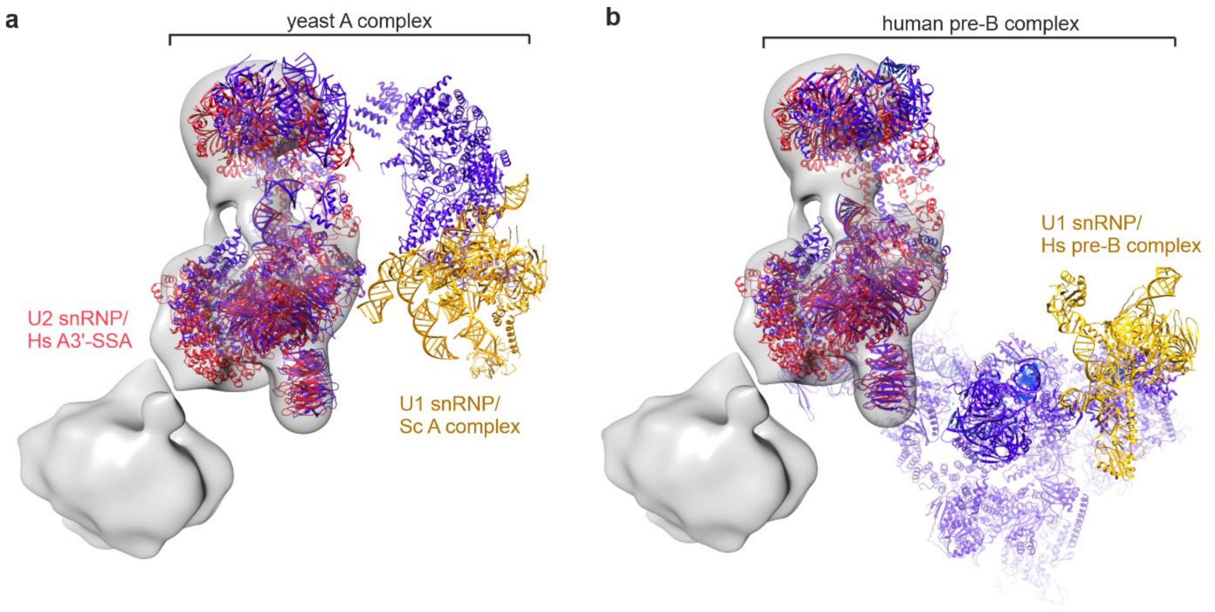

Supplementary Figure 5. Comparison between A3'-SSA, yeast A, and the human pre-B complexes. a Superposition between A3'-SSA (red) and the yeast A complex (PDB 6g90, r.m.s.d. 3.8 Å for 952 equivalent residues from SF3B). U1 snRNP is depicted in gold, whereas the other A complex subunits are coloured in blue. **b** Structural superposition between A3'-SSA (red) and the human pre-B complex (PDB 6qx9, r.m.s.d. 0.837 Å for 1239 equivalent residues from SF3B). U1 snRNP is depicted in gold, whereas the other pre-B complex subunits are coloured in blue. The overall cryo-EM map of A3'-SSA (map M1) is shown in gray.

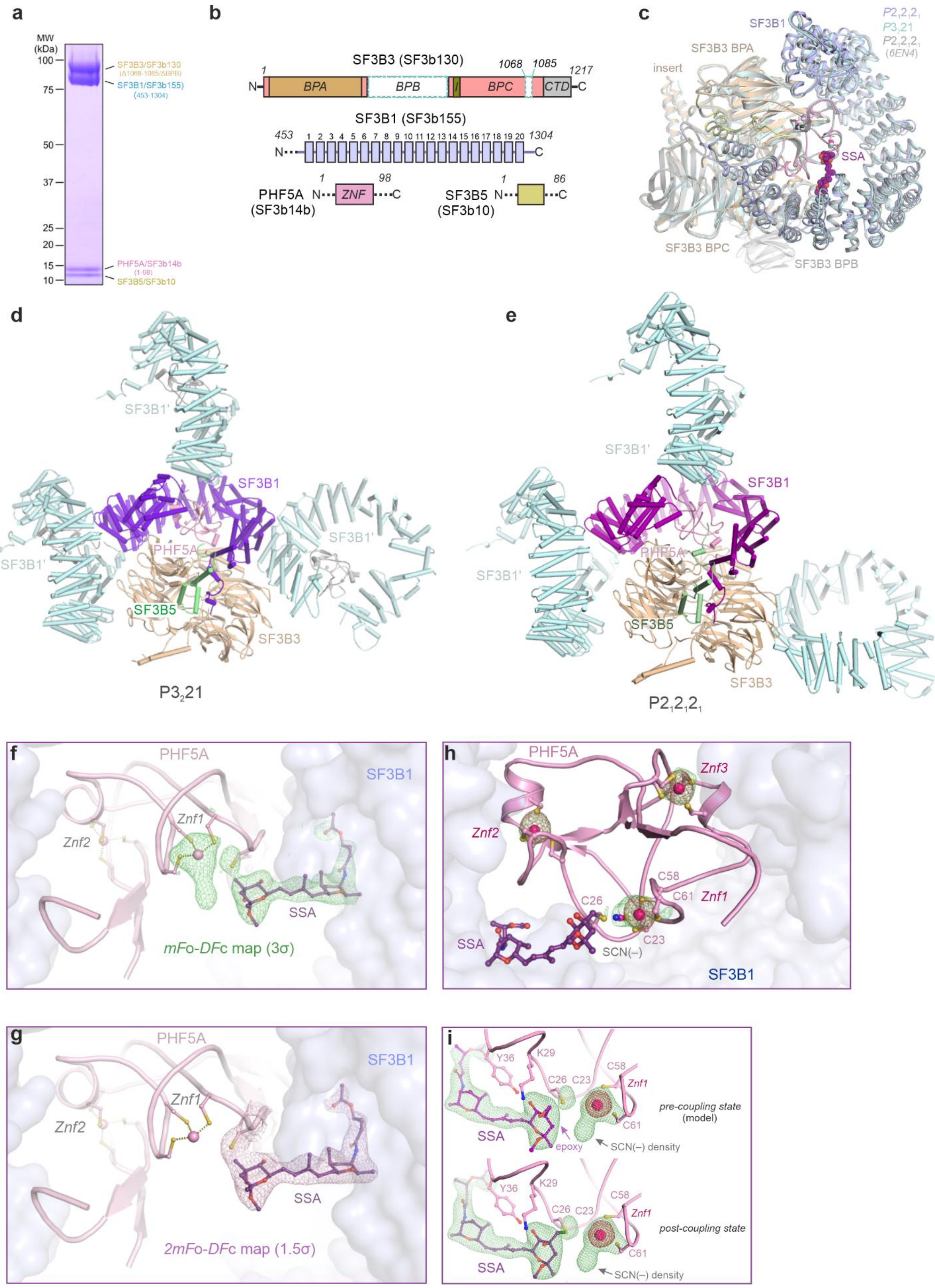

a
b
c
d
e
f
g
h
i
SF3B3 (SF3b130)
BPA
BPB
BPC
CTD
SF3B1 (SF3b155)
PHF5A
(SF3b14b)
ZNF
SF3B5
(SF3b10)
SF3B1
SF3B3 BPA
insert
SSA
SF3B3 BPC
SF3B3 BPB
SF3B1'
PHF5A
SF3B5
SF3B3
P3221
P212121
Znf1
Znf2
Znf3
mFo-DFc map (3σ)
2mFo-DFc map (1.5σ)
SCN(–)
pre-coupling state
(model)
post-coupling state
SCN(–) density
epoxy

Supplementary Figure 6. Crystallization and structure determination of a truncated SF3B core lacking the BPB domain of the SF3B3 subunit (SF3B$^{\Delta BPB}$) in complex with SSA. **a** SDS-PAGE analysis of the purified SF3B$^{\Delta BPB}$ lacking the BPB domain of the SF3B3 subunit. The recombinant SF3B$^{\Delta BPB}$ complex was purified to homogeneity from insect cells at least three times. Source data are provided as a Source Data File. **b** Domain composition schematic of the SF3B$^{\Delta BPB}$ complex. **c** Structural superposition of SF3B core complexes co-crystallized with small-molecule splicing modulators in different crystal forms. **d**-**e** Crystal packing of the SF3B$^{\Delta BPB}$ complex in two different lattices. Importantly, SF3B1 exhibits the same "open" conformation in the trigonal and orthorhombic crystal forms. **f** Co-crystal structure of SF3B$^{\Delta BPB}$ in complex with SSA at 2.3 Å resolution. The residual *m*Fo-*D*Fc map is contoured around the ligand at 3 σ level. The continuous density between C26-PHF5A and SSA is consistent with the formation of a covalent bond. **g** Co-crystal structure of SF3B$^{\Delta BPB}$ in complex with SSA at 2.3 Å resolution oriented and color-coded as in **f**. The *2m*Fo-*D*Fc map is contoured around the ligand at 1.5 σ level. **h** Anomalous difference maps (dark red mesh, contoured at 6 σ level) of the three zinc zinger motifs of PHF5A. The strong anomalous peaks are consistent with the presence of all three zinc atoms in the SF3B$^{\Delta BPB}$-SSA co-crystals. The residual *m*Fo-*D*Fc maps of the zinc ions (green mesh, contoured at 3 σ level) are shown for comparison. **i** Reaction of the epoxy group of SSA with C26-PHF5A does not result in the dissociation of Znf1's zinc ion. The residual *m*Fo-*D*Fc map of Znf1 (green mesh, 3 σ level) shows that the modified C26 residue is no longer in coordination distance from the zinc ion. The freed zinc coordination shell is, likely, occupied by a thiocyanate ion (SCN(−)) present in the crystallization solution. The anomalous difference map is coloured as in **h**.

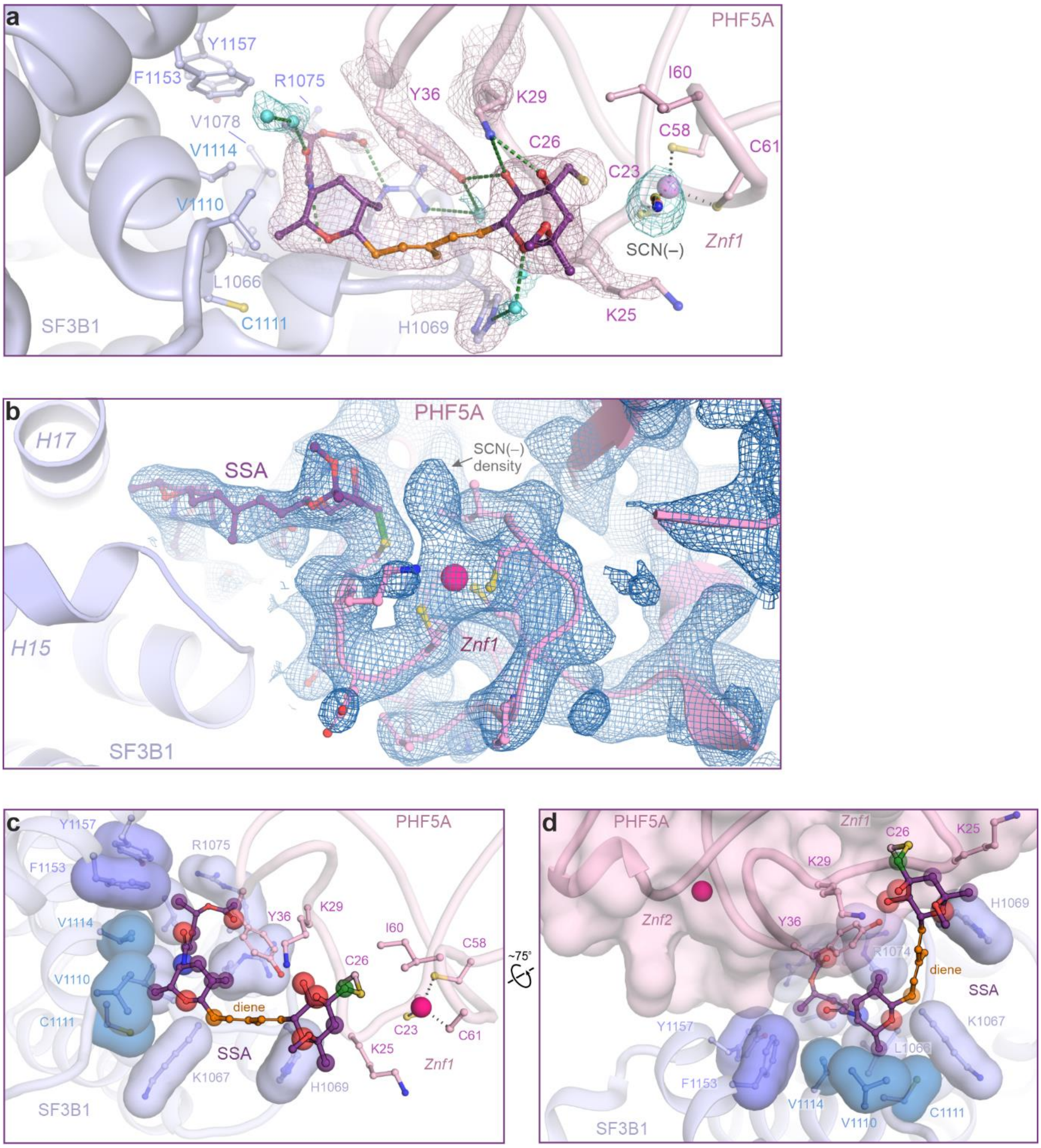

a
PHF5A
Y1157
F1153
R1075
V1078
V1114
V1110
L1066
C1111
SF3B1
H1069
Y36
K29
C26
I60
C58
C61
C23
SCN(–)
Znf1
K25
b
PHF5A
H17
SSA
SCN(–)
density
H15
Znf1
SF3B1
c
Y1157
R1075
PHF5A
F1153
V1114
Y36
K29
I60
C58
V1110
C26
diene
C1111
SSA
C23
C61
K25
K1067
H1069
Znf1
SF3B1
~75°
d
PHF5A
Znf1
C26
K25
K29
H1069
Znf2
Y36
R1074
diene
SSA
Y1157
L1066
K1067
F1153
V1114
V1110
C1111
SF3B1

Supplementary Figure 7. Crystal structure of a truncated SF3B$^{\Delta BPB}$ in complex with SSA. **a** Molecular recognition of SSA by the composite SF3B1-PHF5A binding tunnel. The electron density map (2*m*Fo-*D*Fc, 1.5 σ) of the ligand (purple mesh) is contoured around SSA. SF3B1 and PHF5A residues are shown as sticks, while solvent molecules are shown as spheres and colored in cyan. Polar contacts between SF3B1-PHF5A and the splicing modulator are shown as dashed green lines. **b** Composite omit map of PHF5A-SSA (2*m*Fo-*D*Fc, contoured at 1.0 σ) showing the local reconfiguration of the Znf1 zinc cluster in the orthorhombic crystal form of SF3B$^{\Delta BPB}$. The covalent bond between the C26 residue of PHF5A and SSA is colored in green. **c-d** Structural organization of the SSA binding pocket depicted in two different orientations. SF3B1's residues belonging to the HEAT repeats H15-H17 are shown in surface representation and coloured in light blue (H15 repeat), dark blue (H16 repeat), and slate (H17 repeat), respectively. PHF5A's residues lining the SSA binding site are coloured in pink and depicted as sticks. SSA's chemical groups involved in hydrophobic interactions and polar contacts with SF3B1 and PHF5A are shown as transparent spheres of different sizes. The diene moiety of the ligand is coloured in orange.

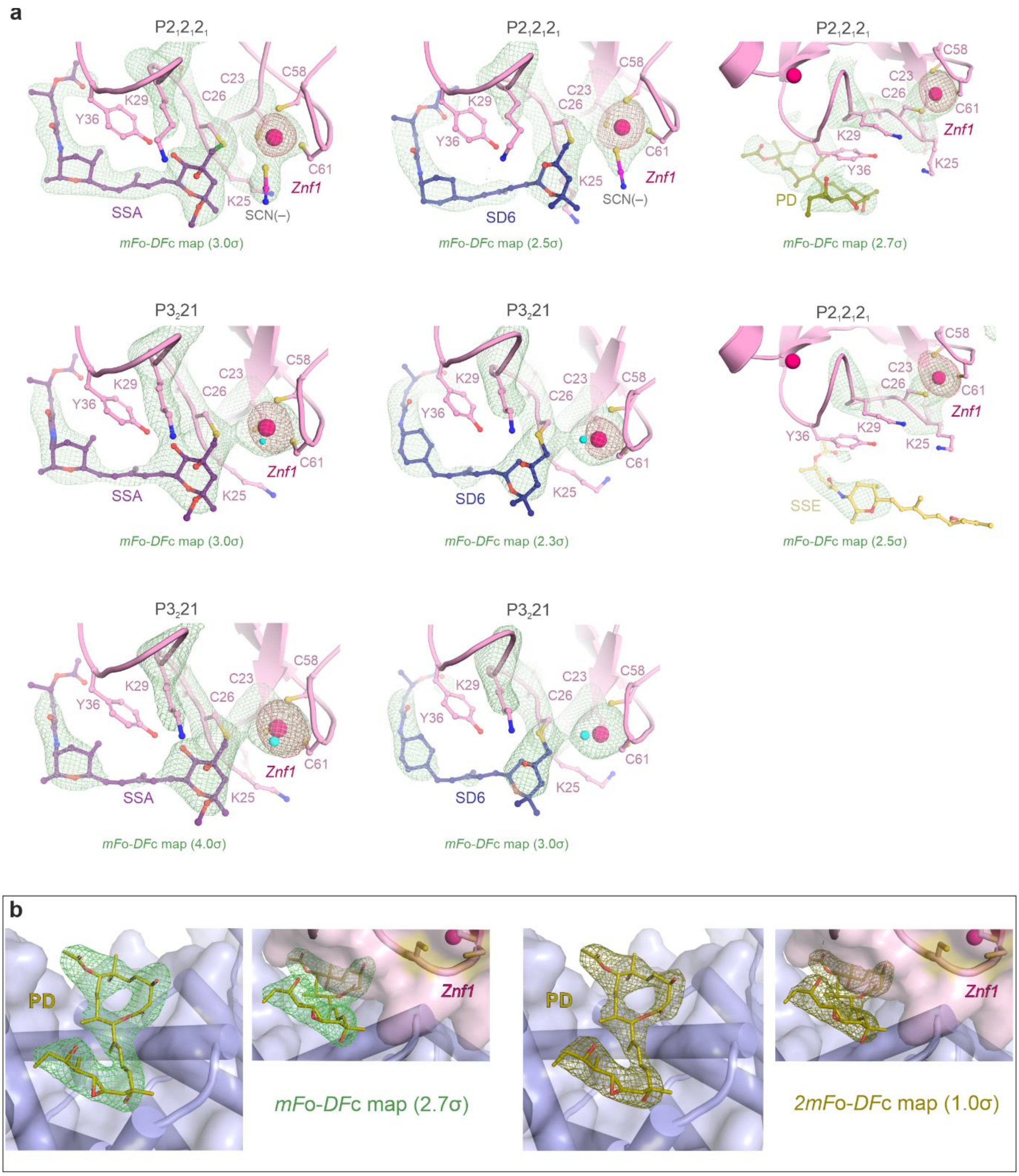

Supplementary Figure 8. Comparison between different splicing modulators. a PHF5A's zinc cluster structure in the presence of different splicing modulatory compounds. The residual *m*Fo-*D*Fc electron density map (green mesh) is displayed at the indicated contour level around the SF3B ligands and residues 23-29 of PHF5A. An anomalous difference map (dark red mesh) is contoured around Znf1's zinc ion. **b** Crystal structure of SF3B$^{\Delta BPB}$ in complex with pladienolide D (PD). The residual *m*Fo-*D*Fc (green mesh, 2.7 σ) and 2*m*Fo-*D*Fc (dark olive, 1.0 σ) maps are contoured around the ligand, and the PD binding pocket is depicted in two different orientations. In spite of targeting the same pocket, pladienolide D differs substantially from FR901464 analogues in its overall structure and distance to Znf1.

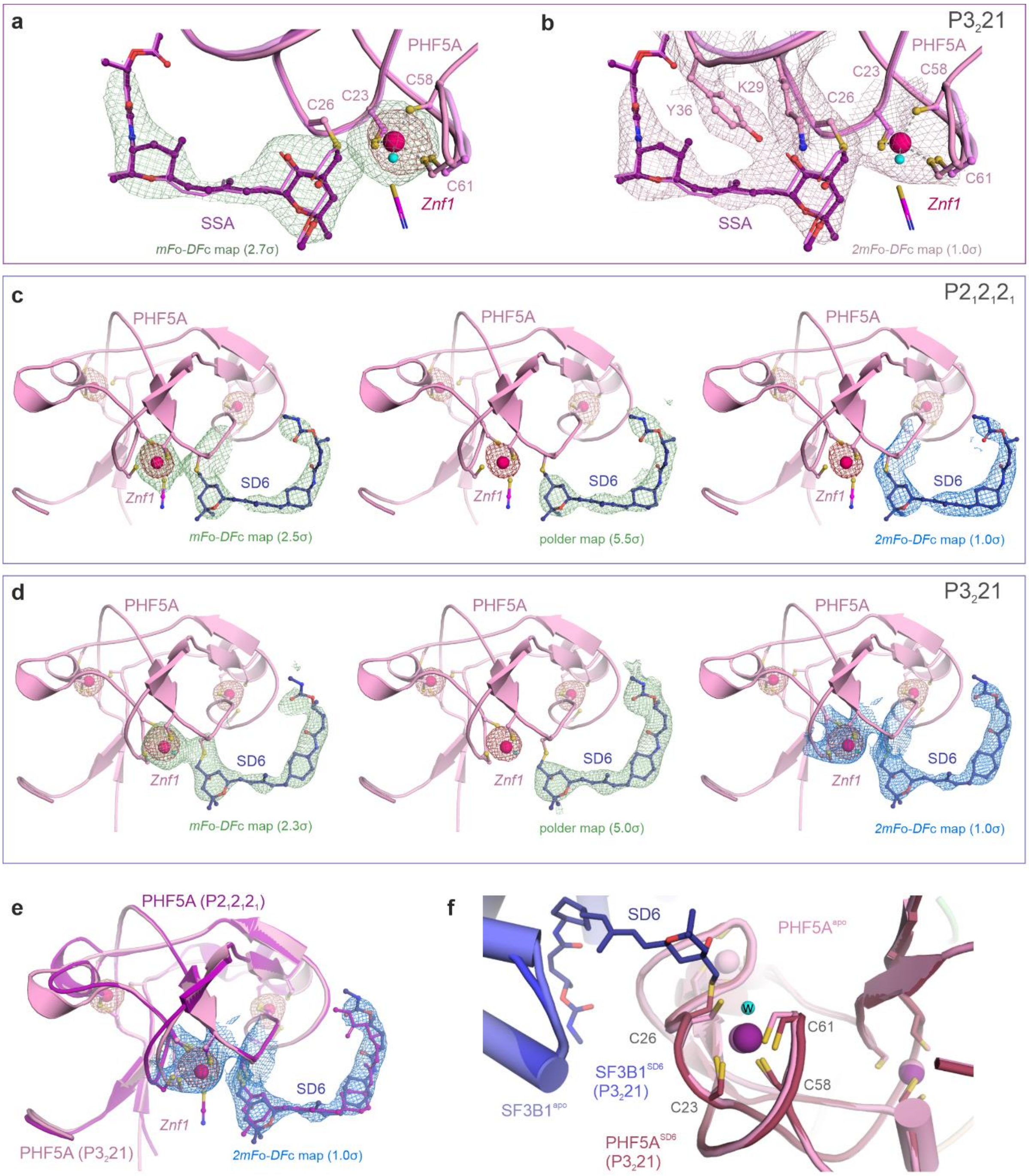
a
b
P3$_2$21
PHF5A
C58
C23
C26
C61
Znf1
SSA
mFo-DFc map (2.7σ)
Y36
K29
2mFo-DFc map (1.0σ)
c
P2$_1$2$_1$2$_1$
SD6
mFo-DFc map (2.5σ)
polder map (5.5σ)
2mFo-DFc map (1.0σ)
d
P3$_2$21
mFo-DFc map (2.3σ)
polder map (5.0σ)
e
PHF5A (P2$_1$2$_1$2$_1$)
PHF5A (P3$_2$21)
f
PHF5A^{apo}
SF3B1^{SD6} (P3$_2$21)
SF3B1^{apo}
PHF5A^{SD6} (P3$_2$21)
W

Supplementary Figure 9. Spliceostatin A (SSA) and sudemycin D6 (SD6) in complex with the SF3B$^{\Delta BPB}$ crystallize both in the presence or absence of thiocyanate in two distinct space groups. a-b Electron density maps of SSA crystallized in complex with SF3B$^{\Delta BPB}$ in an alternative trigonal form ($P3_221$). The residual *m*Fo-*D*Fc map (green mesh) is contoured at 2.7 σ around SSA, while the 2*m*Fo-*D*Fc map (purple mesh) and the anomalous difference map (dark red mesh) are contoured at 1.0 σ and 5.0 σ levels, respectively. PHF5A-SSA from the SF3B$^{\Delta BPB}$'s higher resolution model (light magenta) is superimposed onto the complex's alternative crystal form. As in the case of the orthorhombic crystal form, SSA covalently modifies the C26 residue of PHF5A. However, zinc ion's freed coordination shell is, likely, replaced by a solvent molecule (cyan) rather than a thiocyanate ion. **c-d** Electron density maps of sudemycin D6 (SD6) bound to SF3B$^{\Delta BPB}$ in two alternative crystal forms. The residual *m*Fo-*D*Fc (green mesh), polder (green mesh), and 2*m*Fo-*D*Fc (blue mesh) maps are contoured around SD6. The anomalous difference maps (dark red mesh) for PHF5A's three zinc ions are contoured at 6.0 σ level. **e** Superposition of PHF5A-SD6 from SF3B$^{\Delta BPB}$'s two alternative crystal forms. The 2*m*Fo-*D*Fc electron density map (1.0 σ level) is contoured around SD6 and the Znf1's zinc ion in the trigonal form crystal structure ($P3_221$). As in the case of the SF3B$^{\Delta BPB}$-SSA structures, a local change in the Znf1 zinc cluster structure, compared to the complex's apo state, is observed in the orthorhombic form ($P2_12_12_1$), but not in the trigonal crystals ($P3_221$). **f** Structural alignment between PHF5A-SD6 from the trigonal crystal form ($P3_221$) and the complex's apo state. Compared to the orthorhombic crystal form, it is likely that a solvent molecule (w, cyan) occupies the freed zinc coordination shell.

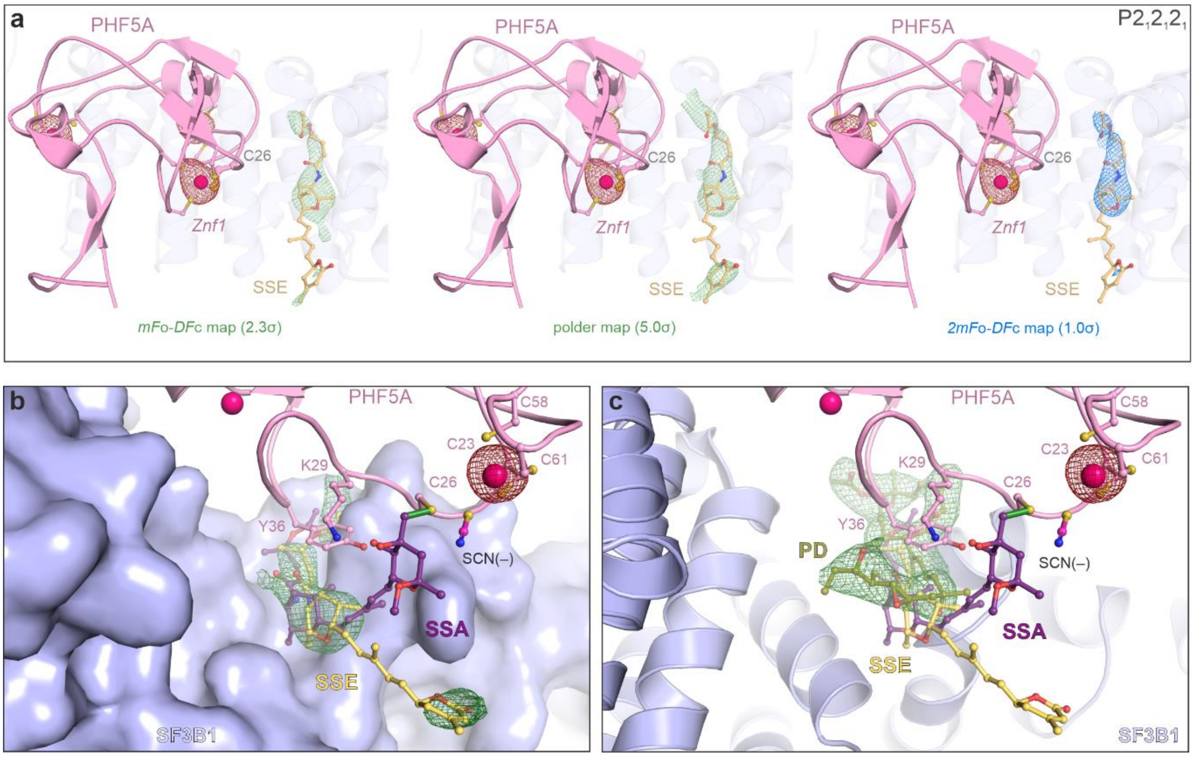

Supplementary Figure 10. Crystal structure of spliceostatin E (SSE) in complex with SF3B$^{\Delta BPB}$. a Crystal structure of SF3B$^{\Delta BPB}$ in complex with SSE. The residual *m*Fo-*D*Fc (green mesh, 2.5 σ), polder (5.0 σ), and 2*m*Fo-*D*Fc (blue, 1.0 σ) electron density maps are displayed around SSE. An anomalous difference map (dark red, 6.0 σ) is contoured around PHF5A's three zinc ions. Note the presence of ligand density in the SF3B1-PHF5A tunnel corresponding to the distal half of SSE; in contrast, density for the proximal moiety is lacking, likely, because of its relative flexibility in the absence of covalent coupling to C26-PHF5A. **b** Superposition between the SF3B$^{\Delta BPB}$-SSA and SF3B$^{\Delta BPB}$-SSE crystal structures. The polder electron density map (green mesh, 5.0 σ) is contoured around SSE. **c** Superposition between SSA, SSE, and PD crystal structures in complex with SF3B$^{\Delta BPB}$. The residual *m*Fo-*D*Fc map (green mesh, 2.7 σ) is displayed around PD.

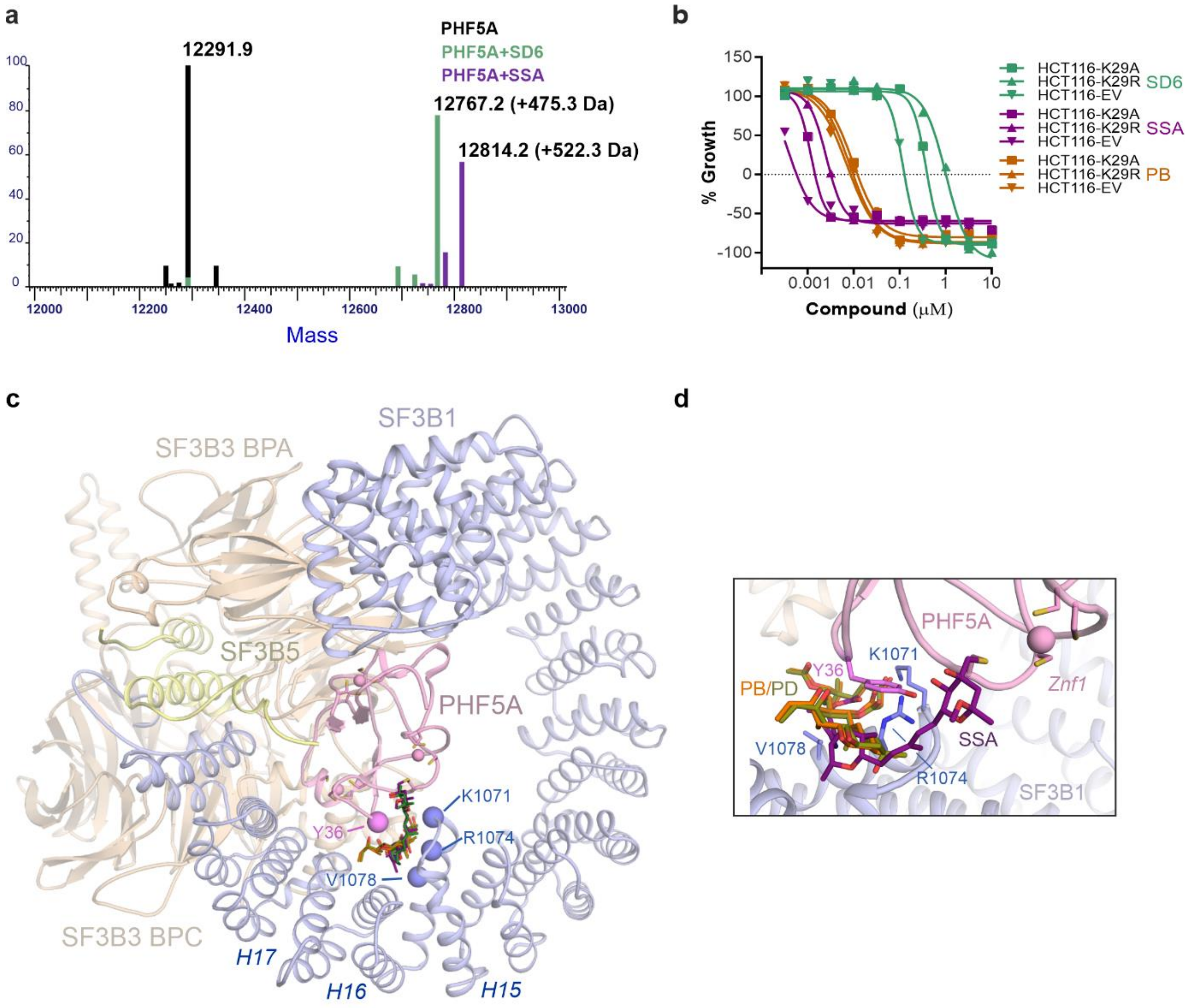

Supplementary Figure 11. Characterization of the covalent coupling of SSA/SD6 to PHF5A by mass spectrometry and viability assays. a Intact mass spectrometry analysis of the minimal SF3B core complex, alone or in the presence of the splicing modulators SSA or SD6. Note the increment in the mass of PHF5A with the expected mass of coupled compounds which shows that the covalent coupling to PHF5A also occurs in solution. The ~99% conversion to the mono-alkylated adduct is consistent with a 1:1 binding stoichiometry, as observed in the crystal structures of SF3B$^{\Delta BPB}$ in complex with SSA/SD6. **b** Cell growth inhibition assays of HCT116 cells harboring the K29A and K29R point mutations in PHF5A. EV denotes the empty vector control (see also the Methods section). Error bar indicates SEM, n=2. Source data are provided as Source Data file. **c-d** Superposition of SSA, SD6, PB (PDB 6EN4), and PD modulators in complex with the SF3B core. Structures were aligned using the PHF5A subunit as a reference. SF3B residues whose substitutions confer resistance to splicing modulation are depicted as spheres (**c**) or sticks (**d**)

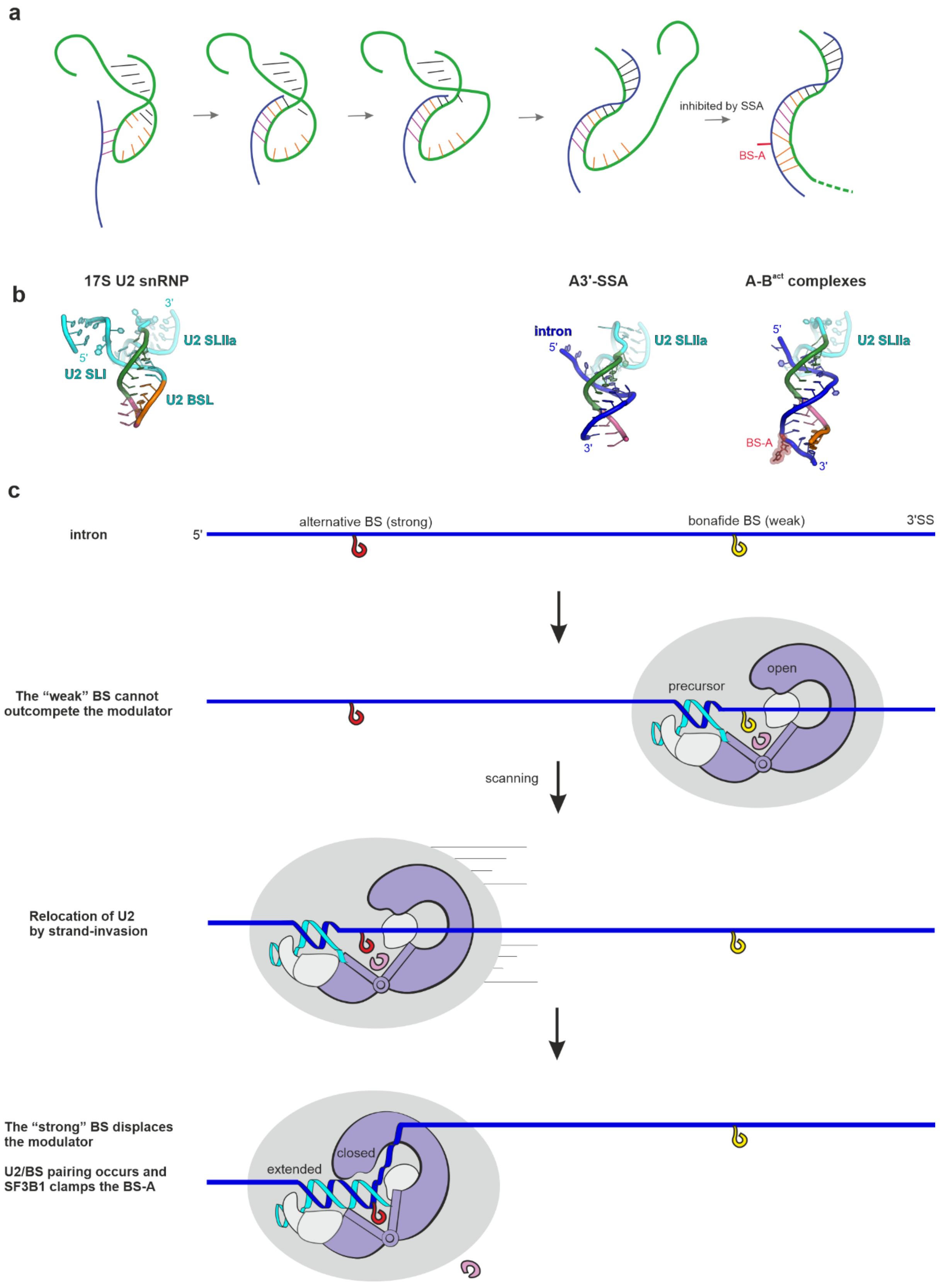

a
inhibited by SSA
BS-A
b
17S U2 snRNP
3'
5'
U2 SLIIa
U2 SLI
U2 BSL
A3'-SSA
intron
5'
U2 SLIIa
3'
A-B^act complexes
5'
U2 SLIIa
BS-A
3'
c
intron
5'
alternative BS (strong)
bonafide BS (weak)
3'SS
The "weak" BS cannot outcompete the modulator
open
precursor
scanning
Relocation of U2 by strand-invasion
The "strong" BS displaces the modulator
U2/BS pairing occurs and SF3B1 clamps the BS-A
closed
extended

Supplementary Figure 12. Model of intron selection by a toehold-mediated strand invasion mechanism. a Starting from an internal toehold, the intron invades the U2-BSL, eventually leading to its unwinding and the formation of a precursor U2/intron duplex. The complete pairing of the BS to U2 snRNA results in the formation of the extended U2/intron helix, where the reactive BS-A is bulged out from the duplex. **b** U2 snRNA and U2-intron structures from the cryo-EM models of different spliceosomal complexes. The structures are positioned below the corresponding intermediates in the panel **a** schematic. The available structural data supports the idea that, while allowing BSL's unwinding, SSA interferes with the formation of the extended U2/intron duplex. **c** A strand-invasion model may enable the relocation of the U2 snRNP to alternative, upstream BSs, when the bonafide BS is "weak" and cannot outcompete the modulators. The U2 model is depicted as in Figure 7 and is simplified for clarity's sake.

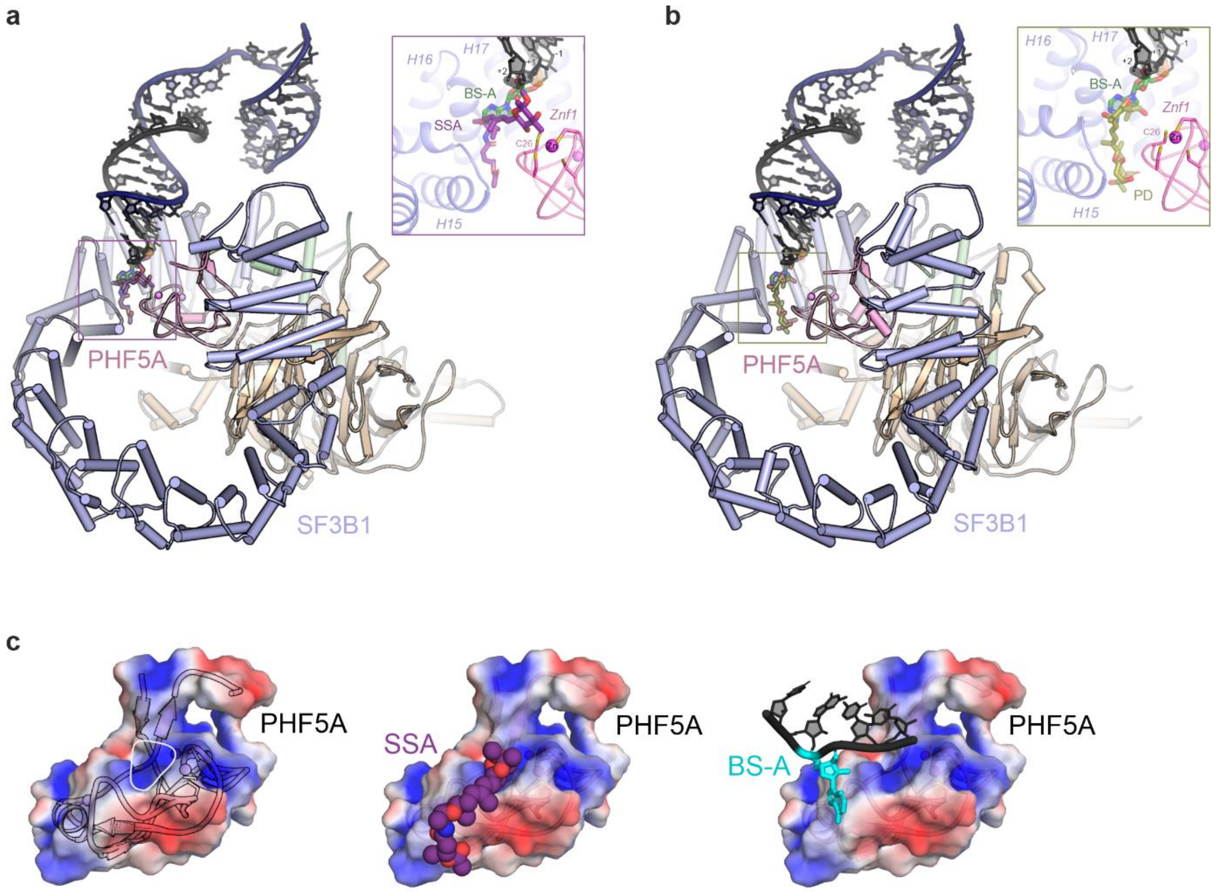

Supplementary Figure 13. FR901464 analogues, pladienolides, and the branch site adenosine bind the hinged pocket of SF3B1 in a mutually exclusive manner. a-b Covalent and non-covalent modulators target the same functional state of the SF3B complex, corresponding to its open conformation. Modulators' binding to the SF3B1-PHF5A pocket interferes with the formation of the extended U2/intron duplex and with the accommodation of the bulged BS-A. The U2/intron extended duplex was modeled into the SF3B core crystal structures as previously described (Cretu et al., 2018) and is based on the cryo-EM structure of the human B^{act} complex (Haselbach et al., 2018). **c** Surface charge distribution of PHF5A's RNA-binding interface. The electrostatic potential (+3 kT/e (blue) to -3 kT/e (red)) was mapped onto the solvent excluded surface of PHF5A with APBS and PyMOL ver. 2.4.1. Notably, the epoxy-tetrahydropyran moiety of SSA, which is covalently linked to C26-PHF5A in $SF3B^{\Delta BPB}$-SSA structure, occupies a positively charged patch on the surface of PHF5A's RNA binding side. The covalent coupling of the epoxy-tetrahydropyran ring to C26-PHF5A appears to reduce the basic character of Znf1's microenvironment, where the K25-PHF5A and K29-PHF5A residues are located. In fully assembled spliceosomes, K25-PHF5A and K29-PHF5A together with Y36-PHF5A participate in recognition of the BS-A and its proximal regions. This raises the possibility that SSA not only acts by trapping the BS-A binding pocket in an "open" conformation, as pladienolide modulators do, but may also alter the electrostatic complementarity between the BS/U2 helix and SF3B.

Supplementary Tables

Supplementary Table 1 Cryo-EM data collection, refinement, and validation statistics

	**Overall map** (map M1)	**U2 17S** (map M4)	**U2 5' module** (EMD-12994) (PDB 7ONB)
**Data collection and processing**			
Electron gun	XFEG		
Detector	K3		
Magnification	81000		
Energy filter slit width (eV)	20.0		
Voltage (kV)	300.0		
Flux on detector (e^-/pix/sec)	22.86		
Electron exposure on sample (e^-/A^2)	41.41		
Target defocus range (µm)	1.0-2.5		
Calibrated pixel size (Å)	1.05		
Symmetry imposed	C1		
Collected movies (no.)	10494		
Initial particle images (no.)	1052001		
Final particle images (no.)	111972	19233	78262
Map resolution at FSC=0.143 (Å)	8.40	10.8	3.07
Map resolution range (Å)			2.9-8.2
**Refinement**			
Initial model used (PDB code)			7B9C, 6FF4, 6FF7
Model resolution (Å)			3.4
Model resolution at FSC=0.5 (Å)			3.3
Map sharpening B factor ($Å^2$)			-54
Model composition			
Non-hydrogen atoms			20142
Protein residues			2568
RNA nucleotides			46
Ligands			1 (SSA)
Waters			-
Ions			5 (5 x Zn^{2+})
B factors ($Å^2$)			
Protein			122.23
Nucleotide			231.68
Ligand			115.18
Water			-
R.m.s. deviations			
Bond lengths (Å)			0.005
Bond angles (º)			0.899
**Validation**			
Molprobity score			1.54
Clashscore			5.59
Rotamer outliers (%)			0.00
Cβ outliers (%)			0.00
CaBLAM outliers (%)			1.68
Ramachandran plot			
Favored (%)			96.45
Allowed (%)			3.55
Disallowed (%)			0.00

Supplementary Table 2 Data collection, refinement, and validation statistics of the co-crystal structures of SF3B^{ΔBPB} in complex with SSA and PD

	**SF3B^{ΔBPB}-SSA (form I)** PDB 7B9C	**SF3B^{ΔBPB}-SSA (form II)** PDB 7B0I	**SF3B^{ΔBPB}-SSA (form I)** Zn K-edge	**SF3B^{ΔBPB}-PD (form I)** PDB 7B91
**Data collection**				
Space group	$P2_12_12_1$	$P3_221$	$P2_12_12_1$	$P2_12_12_1$
Wavelength (Å)	1.0	1.0	1.281	1.023
Cell dimensions				
a, *b*, *c* (Å)	108.57, 111.07, 248.76	108.50, 108.50, 358.94	107.39, 110.26, 248.64	106.07, 109.80, 251.94
α, β, γ (º)	90.0, 90.0, 90.0	90.0, 90.0, 120.0	90.0, 90.0, 90.0	90.0, 90.0, 90.0
Resolution (Å)	49.75-2.3 (2.34-2.3)*	49.41-2.95 (3.05-2.95)*	49.73-2.6 (2.65-2.6)*	50.33-2.81 (2.87-2.81)*
R_{meas} (%)	9.7 (>100)	11.9 (>100)	13.3 (>100)	9.5 (>100)
$I/\sigma I$	18.5 (0.7)	17.6 (0.6)	15.8 (1.1)	16.5 (1.0)
$CC_{1/2}$ (%)	100.0 (32.9)	100 (40.2)	99.9 (54.3)	99.9 (47.4)
Completeness (%)	100 (99.5)	99.9 (99.7)	99.6 (98.7)	100.0 (99.6)
Redundancy	13.6 (13.3)	19.8 (19.6)	13.5 (13.0)	12.9 (12.6)
**Refinement**				
Resolution (Å)	49.44 - 2.4 (2.49-2.4)	43.73-3.0 (3.11-3.0)		50.33-3.0 (3.11-3.0)
No. reflections	118066 (11647)	50086 (4845)		59727 (5890)
R_{work}/R_{free}	0.216/0.248 (0.350/0.375)	0.213/0.243 (0.385/0.413)		0.210/0.255 (0.341/0.378)
No. Atoms				
Protein	14887	14771		14733
Ligand/ion	43	40		42
Water	137	1		1
B-factors (Å^2)				
Protein	74.70	119.22		96.34
Ligand/ion	59.77	97.7		80.76
Water	62.19	86.21		76.69
R.m.s. deviations				
Bond lengths (Å)	0.003	0.002		0.003
Bond angles (º)	0.57	0.51		0.57
Ramachandran plot (%)	96.36/3.59/0.05	95.19/4.64/0.16		95.78/4.22/0

*Statistics for the highest-resolution shell are shown in parentheses.

Supplementary Table 3 Data collection, refinement, and validation statistics of the co-crystal structures of $SF3B^{\Delta BPB}$ in complex with SD6 and SSE

	**$SF3B^{\Delta BPB}$-SD6 (form II)** PDB 7B92	**$SF3B^{\Delta BPB}$-SD6 (form II)** Zn K-edge	**$SF3B^{\Delta BPB}$-SSE (form I)** PDB 7OPI	**$SF3B^{\Delta BPB}$-SD6 (form I)** PDB 7OMF
**Data collection**				
Space group	$P3_221$	$P3_221$	$P2_12_12_1$	$P2_12_12_1$
Wavelength (Å)	1.0	1.282	1.0	1.0
Cell dimensions				
a, b, c (Å)	108.5, 108.5, 358.94	107.3, 107.3, 361.36	104.54, 108.28, 253.20	106.36, 109.85, 253.30
α, β, γ (º)	90.0, 90.0, 120.0	90.0, 90.0, 120.0	90.0, 90.0, 90.0	90.0, 90.0, 90.0
Resolution (Å)	49.41-2.95 (3.05-2.95)*	49.01-3.42 (3.59-3.42)*	49.78-2.9 (2.97-2.90)*	49.03-2.9 (2.97-2.9)*
R_{meas} (%)	11.9 (>100)	25.2 (>100)	8.8 (>100)	9.0 (>100)
$I/\sigma I$	17.6 (0.6)	13.0 (1.4)	17.5 (0.8)	19.1 (0.7)
$CC_{1/2}$ (%)	100 (40.2)	99.9 (52.8)	100 (43.1)	100 (38.9)
Completeness (%)	99.9 (99.7)	99.9 (99.6)	99.9 (98.3)	99.9 (98.9)
Redundancy	19.8 (19.6)	20.7 (21.3)	13.5 (13.8)	13.7 (13.8)
**Refinement**				
Resolution (Å)	46.52-3.0 (3.11-3.0)		48.43-3.1 (3.21-3.1)	48.8-3.0 (3.11-3.0)
No. reflections	49021 (4677)		52968 (5196)	60220 (5926)
R_{work}/R_{free}	0.223/0.258 (0.397/0.414)		0.235/0.277 (0.449/0.445)	0.232/0.266 (0.423/0.447)
No. Atoms				
Protein	14754		14765	14812
Ligand/ion	37		36	40
Water	1		-	-
B-factors (Å^2)				
Protein	102.23		117.56	112.64
Ligand/ion	105.0		113.55	108.18
Water	97.25		-	-
R.m.s. deviations				
Bond lengths (Å)	0.003		0.002	0.003
Bond angles (º)	0.55		0.56	0.61
Ramachandran plot (%)	94.76/5.03/0.22		94.76/4.81/0.43	95.97/3.93/0.11

*Statistics for the highest-resolution shell are shown in parentheses.

Supplementary Table 4 Primer and DNA sequences used in this study

Gene name	Forward (5'-3')	Reverse (5'-3')
SF3B1	CCGAGGAGTCGACCATGAAGTCTGTCAACG	AGGCTCTAGATTACAGGATGTAGTCCAGTTCGTAGC
PHF5A	GATCTCGAGCCATGGCCAAGCACCACCCTGA	GCGCTAGCATTACAGGTCGGTCTTAGAAG
SF3B3 (Δ1068-1085)	TCTCAAAAGGCCGAAGTCATCATGAACTA	GTCCACTTCGTCGTTGGTGTTAGGAGGCA
SF3B3 (ΔBPB)	CAGTCCGTGCCTCAGGACCCTCAGAGAG	GGAGGAAACGGAAACTCTGGAGAAAAGCTGGGAGCCGTGT
A3' gBLOCK	TACTTCCAATCCAATGCAACGCTAGAGGATCCTTGTCGACTAGGTAATACGACTCACTATAGGGCGCAGTAGTCC AGGGTTTCCTTGATGATGTCATACTTATCCTGTCCCTTTTTTTTCCACAGCTCGCGGTTGAGGACAAACTCTTCGC GGTCTTTCCACAGGTAAGTTGGAAGCATGTAGAACCTTGGATCCGATATCCGTACACCATCAGGGTACGAGCTAG CCCATGGCGTACACCATCAGGGTACGACTAGTAGATCTCGTACACCATCAGGGTACGGAATTCTCTAGAGTCGAG TTCTATAGTAATAACATTGGGAAGTGGATAA	
